# Precise Synthesis of ∼1 nm Iridium Nanoclusters as a Catalyst for Efficient Oxygen Evolution

**DOI:** 10.1021/jacs.6c06563

**Published:** 2026-06-15

**Authors:** Tokuhisa Kawawaki, Kotaro Sato, Xiaolin Liu, Maho Kamiyama, Yamato Shingyouchi, Masaki Ogami, D. J. Osborn, Gregory F. Metha, De-en Jiang, Yuichi Negishi

**Affiliations:** † Institute of Multidisciplinary Research for Advanced Materials, 13101 Tohoku University, Katahira 2-1-1, Aoba-ku, Sendai 980-8577, Japan; ‡ Carbon Value Research Center, Research Institute for Science and Technology, 26413 Tokyo University of Science, Kagurazaka, Shinjuku-ku, Tokyo 162−8601, Japan; § Department of Chemical and Biomolecular Engineering, 5718 Vanderbilt University, Nashville, Tennessee 37235, United States; ∥ Department of Chemistry, 1066 Adelaide University, Adelaide, South Australia 5005, Australia

## Abstract

Metal nanoclusters (NCs) with a particle size of approximately 1 nm exhibit potential as highly active catalysts owing to their large specific surface areas and unique electronic structures. However, their precise synthesis in air is predominantly limited to coinage metal (Cu, Ag, and Au) NCs. Consequently, the development of a facile synthesis method for NCs composed of diverse metal elements is highly desirable. Accordingly, we focused on iridium (Ir), which is known for its high catalytic activity in numerous reactions. In this study, we established a precise synthesis method for air-stable Ir NCs and investigated their electrocatalytic activity in the oxygen evolution reaction (OER). We demonstrated that stable Ir_∼15_ NCs can be synthesized employing carbon monoxide and triphenylphosphine as stabilizing ligands. Furthermore, the OER catalysts derived from these Ir_∼15_ NCs as precursors exhibited a 1.5-fold increase in OER activity compared with commercially available Ir catalysts. These findings are anticipated to provide valuable design guidelines for the synthesis of NCs and the development of highly active electrocatalysts using a broad range of metal species.

## Graphical abstract

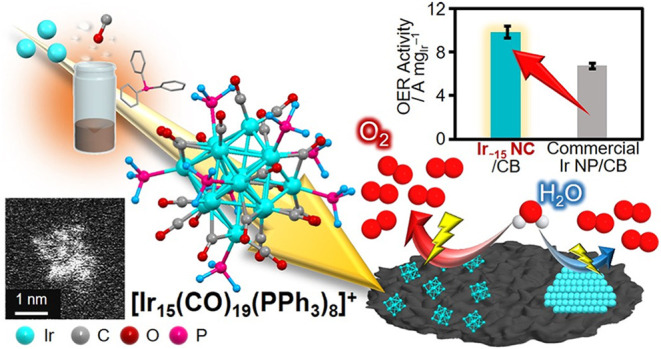

## Introduction

Metal nanoclusters (NCs) exhibit physicochemical properties distinct from their corresponding bulk metals due to quantum size effects.^1–4^ These NCs can be synthesized as compounds with well-defined chemical compositions through the utilization of certain ligands^5–15^ Notably, carbonyl NCs ([M_*n*_(CO)_*m*_]^*z*^) have been investigated since the 1930s, with significant advancements occurring in the 1970s and 1980s, including the discovery of the Chini Pt cluster series.^16–18^ These NCs exhibit superior performances as both thermocatalysts and electrocatalysts compared with their corresponding bulk metals and larger metal nanoparticles (NPs).^19–22^ However, these NCs face a significant challenge: they are highly sensitive to air, making them difficult to synthesize under atmospheric conditions.

Conversely, since the 2000s, it has been demonstrated that atomically precise coinage metal (Cu, Ag, and Au) NCs can be synthesized in air, leading to active research in this area.^23–27^ Specifically, Cu, Ag, and Au NCs stabilized with phosphine (PR), thiolate (SR), etc. ligands exhibit strong metal–ligand bonds, resist oxidation in air, and are easily handled.^28^ However, examples of air-stable metal NCs composed of other metal species synthesized with comparable ease are scarce.^29,30^ Consequently, the catalytic applications of these metal NCs remain restricted.

In our previous studies, we established a synthetic protocol for precise Pt_*n*_(CO)_*m*_(SR)_*l*_ NCs that are readily synthesized in air.^31–33^ Although these NCs can exist with various nuclearities, each species is synthesized with extreme chemical precision (within several atoms). This was realized by the use of both CO and SR ligands, which form relatively strong bonds with Pt. A similar strategy using CO and triphenylphosphine (PPh_3_) ligands has also been demonstrated to yield atomically precise [Pt_17_(CO)_12_(PPh_3_)_8_]^1+/2+^.^34^ While several Pd(0) NCs with well-defined structures have been recently synthesized in air,^35–38^ there are still no reported instances of analogous Ir(0) NCs to the best of our knowledge.^39^

Notably, Ir is recognized as the element that exhibits the highest oxygen evolution reaction (OER) activity in water electrolysis under acidic conditions (Figure S1).^40–42^ Typically, Ir-NP-supported catalysts with particle sizes of several nanometers are employed as electrocatalysts for water electrolysis. While OER catalysts based on less expensive base metals are under development, achieving comparable durability to Ir-based catalysts in acidic solutions remains challenging.^43–47^ Consequently, the substantial Ir usage in current water electrolysis systems hinders their widespread adoption. Therefore, minimizing the Ir content in electrocatalysts for water electrolysis is crucial. The creation of catalysts supporting fine Ir NCs with particle sizes around 1 nm is expected to enhance reaction active site density, leading to more active OER catalysts and reduced Ir consumption (Figure 1).

**Figure 1:**
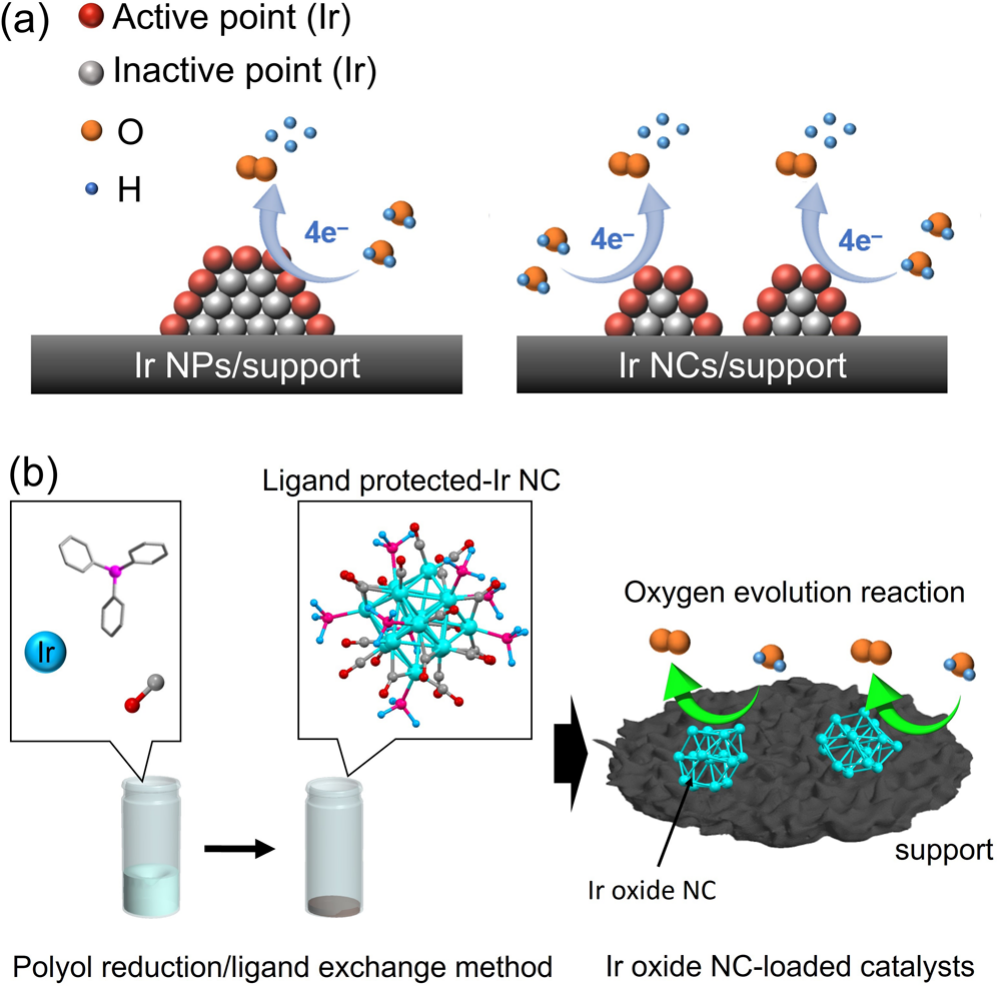
Schematics of this research. Schematics of (a) the size-refinement effects in Ir catalysts and (b) and synthesis of Ir NC and preparation of Ir oxide NC-loaded catalyst and their application to the OER.

To this end, we established a precise synthesis method for air-stable Ir NCs and demonstrated their superior performances as OER catalysts in this work.

## Results and Discussion

### Synthesis of Ir_*n*_(CO)_*m*_(PPh_3_)_*l*_ NCs

The synthesis was conducted using a method combining polyol reduction and ligand exchange. Initially, an ethylene glycol solution containing Iridium­(III) chloride hydrate (IrCl_3_·*x*H_2_O) and sodium hydroxide (NaOH) was heated under air. It is hypothesized that, during this process, CO coordinates to the metal Ir atoms concurrently with the reduction of Ir ions. CO originates from the oxidation of ethylene glycol. Consequently, the resulting Ir NCs are weakly stabilized by CO and OH^–^ in the solution.^31^ Subsequently, by replacing the majority of CO and OH^–^ with triphenylphosphine (PPh_3_), which forms strong Ir–ligand bonds, we converted the Ir NCs into air-stable compounds. Specifically, the solution was cooled to ambient temperature and PPh_3_ was introduced to facilitate ligand exchange on the Ir NCs (Ir_*n*_(CO)_*m*_(PPh_3_)_*l*_ NCs). Ir_*n*_(CO)_*m*_(PPh_3_)_*l*_ NCs were then isolated by removing unreacted PPh_3_, Ir ions, and byproducts from the solution. The matrix-assisted laser desorption/ionization mass spectrometry (MALDI-MS) trace of the Ir_*n*_(CO)_*m*_(PPh_3_)_*l*_ NC mixture shows prominent peaks at *m*/*z* = ∼3400 and ∼4900 (Figure 2a). This suggests the presence of at least two forms of Ir_*n*_(CO)_*m*_(PPh_3_)_*l*_ NCs with distinct stable structures. Subsequently, Ir NCs of the desired size were selectively isolated via solvent extraction (product **1**). The MALDI-MS trace of **1** shows a single intense peak at *m*/*z* = ∼4900, confirming its successful isolation.

**Figure 2:**
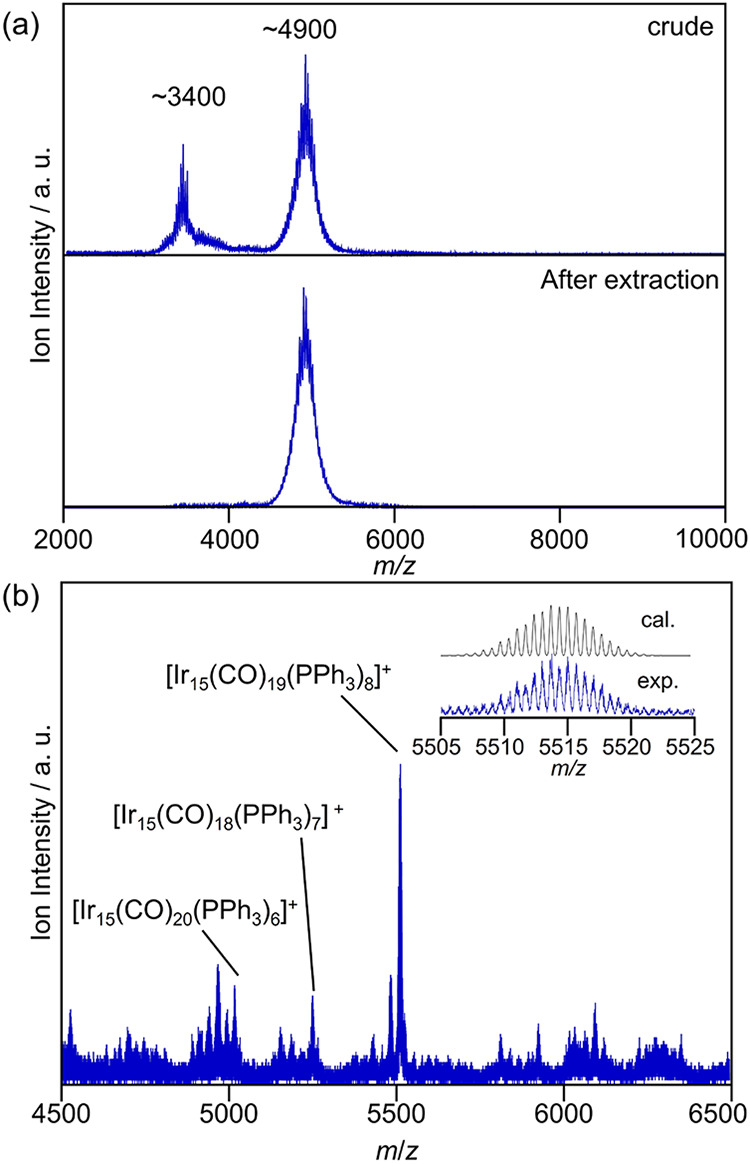
Characterization of 1 by mass spectrometry. (a) MALDI-MS before and after purification of **1** and (b) ESI-MS spectra of **1**.

Metal NCs are susceptible to P–C bond cleavage under laser irradiation, complicating accurate composition determination by MALDI-MS. Therefore, the chemical composition of **1** was elucidated using electrospray ionization mass spectrometry (ESI-MS), which employs gentler ionization processes (Figure 2b). At this stage, ESI-MS spectra revealed that exposing **1** to air for 1 week induced oxygen etching, which further eliminated the unstable Ir_*n*_(CO)_*m*_(PPh_3_)_*l*_ NCs from **1** (Figure S2). The ESI-MS spectrum shows a predominant peak at *m*/*z* = ∼5515, corresponding to [Ir_15_(CO)_19_(PPh_3_)_8_]^+^, with an estimated Ir atom distribution of ±∼2. To further confirm the chemical composition of **1**, Ir NCs were synthesized using diphenyl­(*p*-tolyl)­phosphine (DPTP) instead of PPh_3_. The positive-ion ESI-MS spectrum of the resulting product (**2**) is presented in Figure S3. A strong peak is observed around *m*/*z* = ∼5598, assigned to [Ir_15_(CO)_18_(DPTP)_8_]^+^. This indicates that one CO ligand is displaced by the bulkier DPTP ligand, resulting in the formation of **2**. Since both the PR_3_ and CO ligands are neutral ligands, it is presumed that the electronic change is not significant even if the number of CO ligands is changed. This observation strongly supports the predominant formation of Ir_∼15_ NCs based on stable electronic/geometric structure. The metallic electronic states of Ir and the coordination of PPh_3_ (Figure S4) were also confirmed by X-ray photoelectron spectroscopy (XPS).

Unfortunately, we were unable to obtain a single crystal of product **1** (Figure 3) suitable for X-ray analysis. Therefore, its structure was modeled using density functional theory (DFT) calculations, where PPh_3_ ligands were replaced with PH_3_ to reduce the computational cost. The optimized structure of [Ir_15_(CO)_19_(PH_3_)_8_]^+^ is shown in Figure 3g. The metallic core exhibits a distorted ellipsoidal geometry, consisting of an Ir_13_ cuboctahedral unit capped by two Ir atoms at the top and bottom with relatively long Ir–Ir bonds. Unlike typical 13-atom Au NCs that often adopt icosahedral geometries, this Ir_15_ core features a face-centered cubic (fcc) packing (Figures S5 and S6), closely resembling the Au_13_ core of Au_15_ kernel observed in [Au_23_(SR)_16_]^−^.^48,49^ Such fcc-based frameworks are relatively rare among Group 9 metal clusters protected solely by carbonyl ligands, which typically favor hexagonal close-packed (hcp) or other geometries, as seen in [Ir_14_(CO)_27_]^−^, [Rh_15_(CO)_27_]^3–^ and [Rh_15_(CO)_27_]^3–^.^17,18,50,51^ We infer that the introduction of phosphine ligands, which are sterically bulkier than CO, facilitates the formation of this specific fcc core. The DFT-optimized structure reveals that the PH_3_ and bridging CO ligands preferentially occupy the vertices where steric congestion is minimized. Experimentally, [Ir_15_(CO)_18_(DPTP)_8_]^+^ was formed more readily than [Ir_15_(CO)_19_(DPTP)_8_]^+^ from ESI-MS spectra (Figure S3). This suggests that the increased steric bulk of the methyl groups in the DPTP ligand prevents the coordination of the 19th CO molecule. Indeed, the calculated [Ir_15_(CO)_19_(PH_3_)_8_]^+^ structure contains an Ir site densely coordinated by three bridging CO and PH_3_ groups; such sites are likely prone to CO deficiency due to steric repulsion. Similar ligand-deficient sites caused by steric hindrance have been reported in other transition metal NCs.^52^

**Figure 3:**
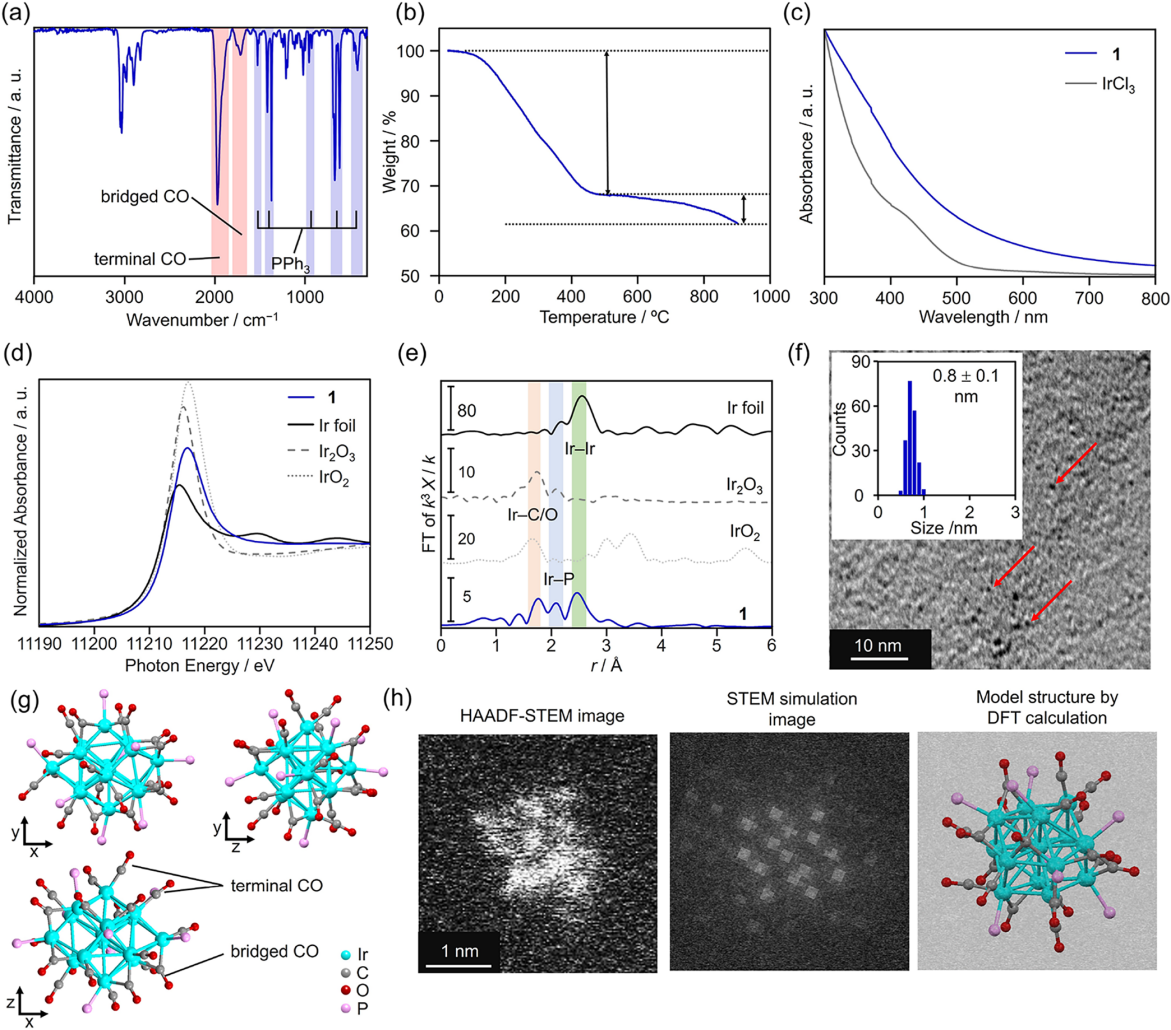
Detailed characterization of 1 by FT-IR, TGA, optical spectroscopy, XANES, FT-EXAFS, and TEM. (a) FT-IR spectrum, (b) TGA curve, (c) optical absorbance spectra, Ir L_3_-edge (d) XANES and (e) FT-EXAFS spectra, (f) TEM image and the resulting size histogram, (g) optimized structure of [Ir_15_(CO)_19_(PH_3_)_8_]^+^ from DFT calculation and (h) HAADF-STEM image, simulated image and model structure of **1**. In (c), the precursor IrCl_3_·*x*H_2_O is also shown for comparison. In (d) and (e), Ir foil, Ir_2_O_3_, and IrO_2_ powder as references are also shown for comparison. The spectrum of **1** was measured at 10 K for analysis with suppressed thermal vibration. The *k* range was set to 3.0–18.0 Å^–1^. In (g) and (h), for better visual clarity, the hydrogen atoms has been simplified.

To explore the existence of other stable NC species, we also attempted to synthesize smaller Ir NCs by reducing the thermal reduction time. This led to the size selection of Ir_*n*_(CO)_*m*_(PPh_3_)_*l*_ NCs, corresponding to the peak around *m*/*z* = ∼3400 observed by MALDI-MS (product **3**; Figure S7a). ESI-MS of **3** shows the presence of a dominant peak assigned to [Ir_6_(CO)_3_(PPh_3_)_8_Cl]^+^ (Figure S7b). A similar study using 2-phenylethanethiolate (PET) as the SR ligand instead of PPh_3_ indicated the formation of Ir_*n*_(CO)_*m*_(PET)_*o*_ NCs (**4**), coprotected by CO and PET (Figure S8).

To further corroborate the presence of CO and PPh_3_ ligands in **1**, Fourier transform infrared spectroscopy (FT-IR) measurements were conducted (Figure 3a). The resulting spectrum shows peaks at 1941 and 1742 cm^–1^, corresponding to terminal and bridged CO ligands, respectively. Additionally, multiple peaks within the 500–1500 cm^–1^ range, attributed to PPh_3_, are observed. These findings confirm the presence of both CO and PPh_3_ in the product.^34^

Furthermore, the thermogravimetric analysis (TGA) curve of **1** is shown in Figure 3b. The first weight loss occurs between 100–400 °C, and the second commences at 580 °C. Based on prior studies and the estimated chemical composition, the first weight loss is attributed to PPh_3_ desorption, while the second corresponds to CO desorption. This two-stage weight loss pattern further supports the presence of CO and PPh_3_ ligands.^34^ Due to the strong affinity of CO for the iridium surface, it is possible that the CO ligands did not completely desorb within the TG measurement range (up to 900 °C), potentially leading to an underestimation of the total weight loss. Moreover, the increased absorption in the visible to near-infrared region of the optical absorption spectrum (Figure 3c) signifies the formation of Ir NCs from the precursor Ir complex and the transition to a continuous electronic structure.

The electronic structure and bonding characteristics of **1** were further investigated using Ir L_3_-edge X-ray absorption fine structure (XAFS) measurements. To minimize bond fluctuations induced by thermal vibrations, the measurements were conducted at 10 K. Figure 3d presents the Ir L_3_-edge X-ray absorption near edge structure (XANES) spectrum. Compared with Ir foil, the white line onset of **1** exhibits a slight shift toward higher energy and with enhanced intensity. This observation is attributed to the discrete electronic levels of the diminutive NCs, suggesting that **1** possesses a cationic electronic structure relative to that of Ir(0). Additionally, the Ir L_3_-edge FT-extended X-ray absorption fine structure (FT-EXAFS) spectrum shows peaks corresponding to Ir–Ir (2.5–2.8 Å), Ir–P (1.9–2.2 Å), and Ir–C (1.5–1.8 Å) bonds (Figure 3e and Table S1). Furthermore, curve fitting analysis of the FT-EXAFS spectrum (Table S1) indicates that **1** exhibits an Ir–Ir bond distance comparable to that of bulk Ir metal (2.71 Å for Ir foil vs. 2.67 Å for **1**). The Ir–C coordination number was determined to be 1.1, suggesting that CO ligands primarily coordinate to the Ir NC as terminal CO, which aligns with the FT-IR findings.

Furthermore, these interpretations align with the particle size (0.8 ± 0.1 nm; Figure 3f) determined by transmission electron microscopy (TEM). The high-angle annular dark-field scanning TEM (HAADF-STEM) images of **1** (Figure 3h) indicates a relatively anisotropic structure,^50,53,54^ a morphology frequently observed in anaerobically synthesized carbonyl NCs such as M_*n*_(CO)_*m*_ NCs. While Ir_*n*_(CO)_*m*_ NCs, including [Ir_4_(CO)_11_]^2–^, [Ir_4_(CO)_10_]^4–^, [Ir_6_(CO)_15_]^2–^, [Ir_8_(CO)_22_]^2–^, [Ir_9_(CO)_20_]^3–^, [Ir_10_(CO)_21_]^2–^, [Ir_14_(CO)_27_]^−^, have been reported,^50,53–56^ air-stable Ir NCs synthesized with comparable ease are unprecedented. These findings underscore the unique characteristics of the Ir_*n*_(CO)_*m*_(PPh_3_)_*l*_ NCs synthesized in this study.

### Preparation of Ir_*n*_ NC-Loaded Catalysts

Carbon black (CB) is extensively employed as a support material for electrocatalysts. Consequently, we attempted to support the synthesized Ir NCs on CB. Initially, **1** was adsorbed onto CB by introducing it into a dichloromethane solution containing **1** (**1**/CB). At this point, a solution of **1** was prepared using inductively coupled plasma mass spectrometry (ICP-MS) such that the Ir concentration was 1 wt % relative to the CB. Analysis of the FT-IR spectrum (Figure 4a) reveals absorption bands corresponding to O=P bonds in **1**/CB, which are absent in **1**. This indicates that during the adsorption of **1** onto CB, a portion of the PPh_3_ ligands in **1** undergo oxidation to O = PPh_3_, simultaneously detaching from the surface of **1** and transferring to CB.^31^ In fact, the P 2p XPS spectrum of **1**/CB revealed a shift toward higher binding energies compared to that of **1**, indicating a more oxidized state of the phosphorus species upon supported (Figure S4). Furthermore, considering that defects in CB contain hydroxyl and carboxyl functionalities, it is postulated that these polar functional groups readily form bonds with the labile Ir atoms exposed following ligand removal.^31^ It is well-established that residual ligands on NCs can diminish catalytic activity by impeding reactant accessibility and hindering electron transfer with CB. Therefore, the majority of ligands were removed from **1** by subjecting the resulting sample to thermal treatment at 300 °C under reduced pressure in an electric furnace (Ir_∼15_ NC/CB). At this stage, the absence of neutral phosphorus species (∼132.2 and ∼131.4 eV) derived from PPh_3_ in the P 2p XPS data further indicates that the PPh_3_ ligands have completely dissociated from the Ir_∼15_ NCs. It should be noted that the atmosphere used during the TG measurements differs from that of the actual catalyst sintering process, particularly regarding the oxygen content. The detached PPh_3_ ligands were found to migrate onto the CB support, where they existed in a highly oxidized state, as evidenced by the P 2p XPS spectra (Figure S4).

**Figure 4:**
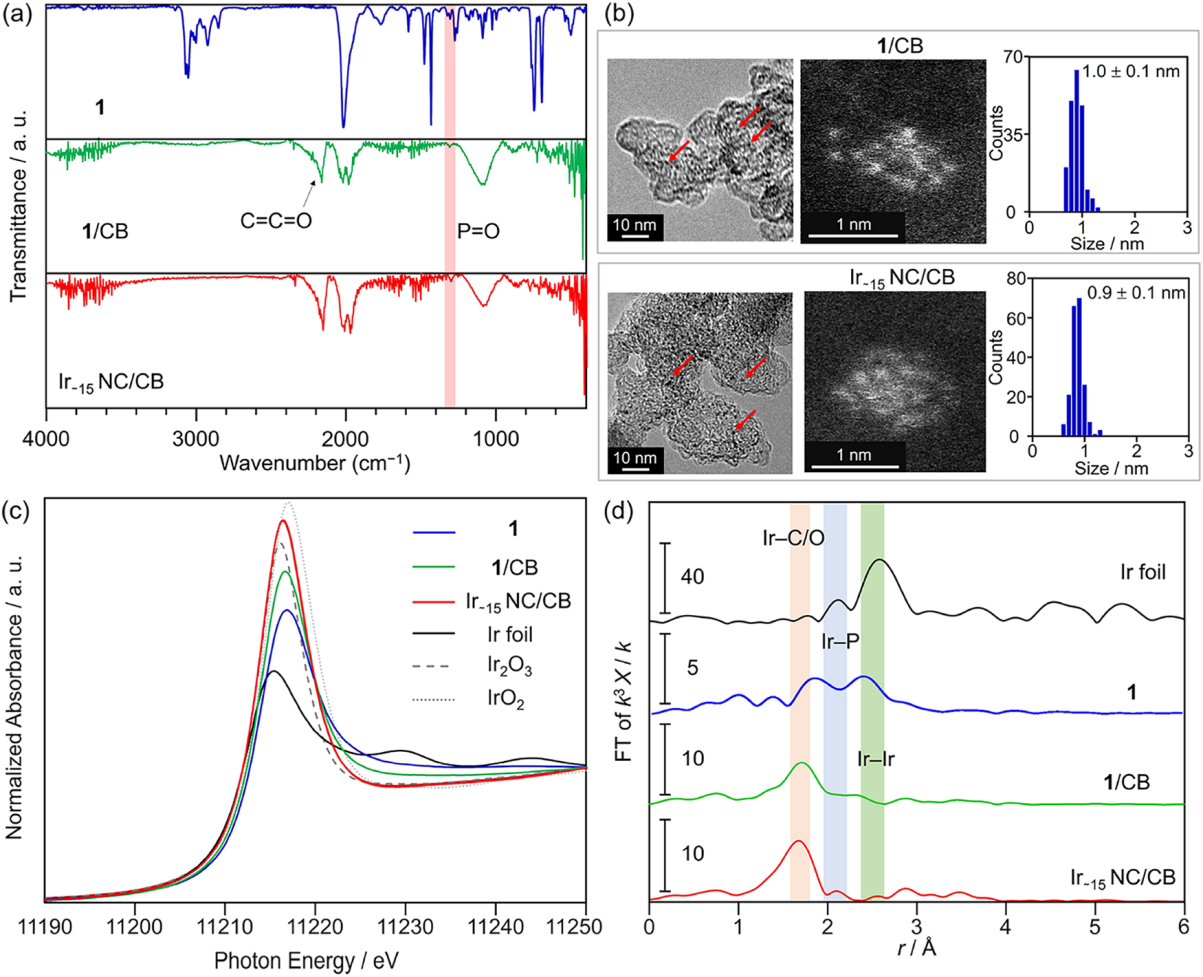
Characterization of 1 before and after the preparation process for the catalyst. (a) FT-IR spectra, (b) TEM images, HAADF-STEM images (and the resulting size histogram), and Ir L_3_-edge (c) XANES and (d) FT-EXAFS spectra of Ir particles of **1** after absorption on CB (**1**/CB) and after calcination of **1**/CB (Ir_∼15_ NC/CB). In (c), Ir foil, Ir_2_O_3_, and IrO_2_ powder as reference are shown for comparison, and In (d), Ir foil is also shown. In (c) and (d), the spectra were measured at room temperature and the *k* range was set to 3.0–14.0 Å^–1^ for comparison. In (d), Ir–C/O scattering at 1.5–1.8 Å includes CO ligands and adsorbed oxygen from air. In (a), the pink region is attributable to the *P* = O vibration.

Figure 4b shows TEM images of the catalysts at each stage of preparation. The Ir particles in **1**/CB, according to TEM, are slightly larger than those for **1**. This is attributed to the Ir particles for Ir_∼15_ NC/CB adopting a flatter morphology due to interactions with the CB support. Furthermore, the Ir particles in the Ir_∼15_ NC/CB obtained after calcination exhibit a particle size nearly identical to that for **1**/CB. This suggests that **1** is strongly immobilized on CB during the adsorption process, minimizing aggregation during calcination of **1**. The strong adsorption of **1** onto the CB support is attributed to the high binding energy between Ir and C, which was further supported by the EXAFS analysis discussed below. Additionally, TEM and HAADF-STEM images confirm the successful support of fine Ir particles (size = 0.9 ± 0.1 nm) on CB (Figures 4b and S9). Based on the TGA curves in Figure 3b, it is inferred that residual ligands persist on the Ir particle surfaces, even after calcination at 300 °C. These residual ligands likely play a pivotal role in suppressing the aggregation of Ir_∼15_ NCs during calcination. As discussed subsequently, excessive ligand retention at lower calcination temperatures results in diminished catalytic activity. Indeed, comprehensive evaluations of Ir_∼15_ NC-supported catalysts calcined at various temperatures indicate that calcination within the 250–350 °C range does not significantly alter particle size but induces subtle modifications in the Ir d-band structure (Figures S9–11).

Subsequently, the electronic state of the supported Ir_∼15_ NCs was determined from the Ir L_3_-edge XANES spectrum depicted in Figure 4c. All measurements were conducted at ambient temperature to replicate the conditions of the electrochemical assessments. The white line intensity of **1**/CB is marginally higher than that of **1**. This observation corroborates the hypothesis that a part of the ligands in **1** desorb and **1** become immobilized on CB during adsorption process. Furthermore, the Ir particles in Ir_∼15_ NC/CB exhibited enhanced oxidation following calcination compared to **1**/CB, displaying an electronic state akin to that of Ir_2_O_3_. This electronic state represents a more oxidized state than that of the commercial Ir NP/CB catalyst. Figure 4d shows the Ir L_3_-edge FT-EXAFS spectra of **1**/CB and Ir_∼15_ NC/CB. In **1**/CB, the Ir–Ir (2.5–2.8 Å) and Ir–P (1.9–2.2 Å) intensities of the bond contribution are diminished relative to those for **1**, while the Ir–C/O bond (1.5–1.8 Å) became stronger, indicating structural alterations upon support on CB. This structural transformation is attributed to the high binding energy between Ir and C, which is consistent with the structural modifications observed in the aforementioned TEM analysis. Additionally, in Ir_∼15_ NC/CB, the Ir–C/O (1.5–1.8 Å) intensity of the bond contribution is augmented, suggesting oxygen adsorption onto the exposed Ir atoms at the surface due to calcination. Concurrently, a significant decrease in the Ir–Ir coordination was detected, indicating that oxygen incorporation into the metal core led to the cleavage of Ir–Ir bonds and a subsequent transformation into an Ir oxide-like NC.

Overall, although structural rearrangements occurred in the Ir_∼15_ NCs due to their interaction with the CB support, the morphology observed in the HAADF-STEM images remained evident even after sintering (Figure S9), suggesting that the anisotropic geometric framework of the Ir_∼15_ NCs was preserved. Thus, we successfully supported Ir_∼15_ NCs onto CB and maintained a particle size comparable to that observed in solution synthesis.

### Oxygen Evolution Activity of Ir_*n*_ NC-Loaded Catalysts

The OER activity of the prepared Ir_∼15_ NC/CB was evaluated under conditions approximating those of practical applications (Figures 5a–c and S12 and Tables S2 and S3). Prior to measurement, the electrode was cleaned by 100 cycles of cyclic voltammetry (CV) in a nitrogen (N_2_) atmosphere, a procedure intended to remove residual ligands and surface organic compounds. Figure S13 illustrates the evolution of CV curves over successive cycles. The results indicate that the electrochemical treatment effectively activates the surface of the Ir catalysts across all samples, facilitating proton adsorption at potentials more negative than +0.4 V vs. RHE. Specifically, commercial Ir NP/CB­(P40A050) catalyst exhibited more pronounced proton adsorption peaks, which is attributed to its higher Ir(0) content. Furthermore, all samples displayed comparable double-layer capacitances, suggesting similar electrochemically active surface areas.

**Figure 5:**
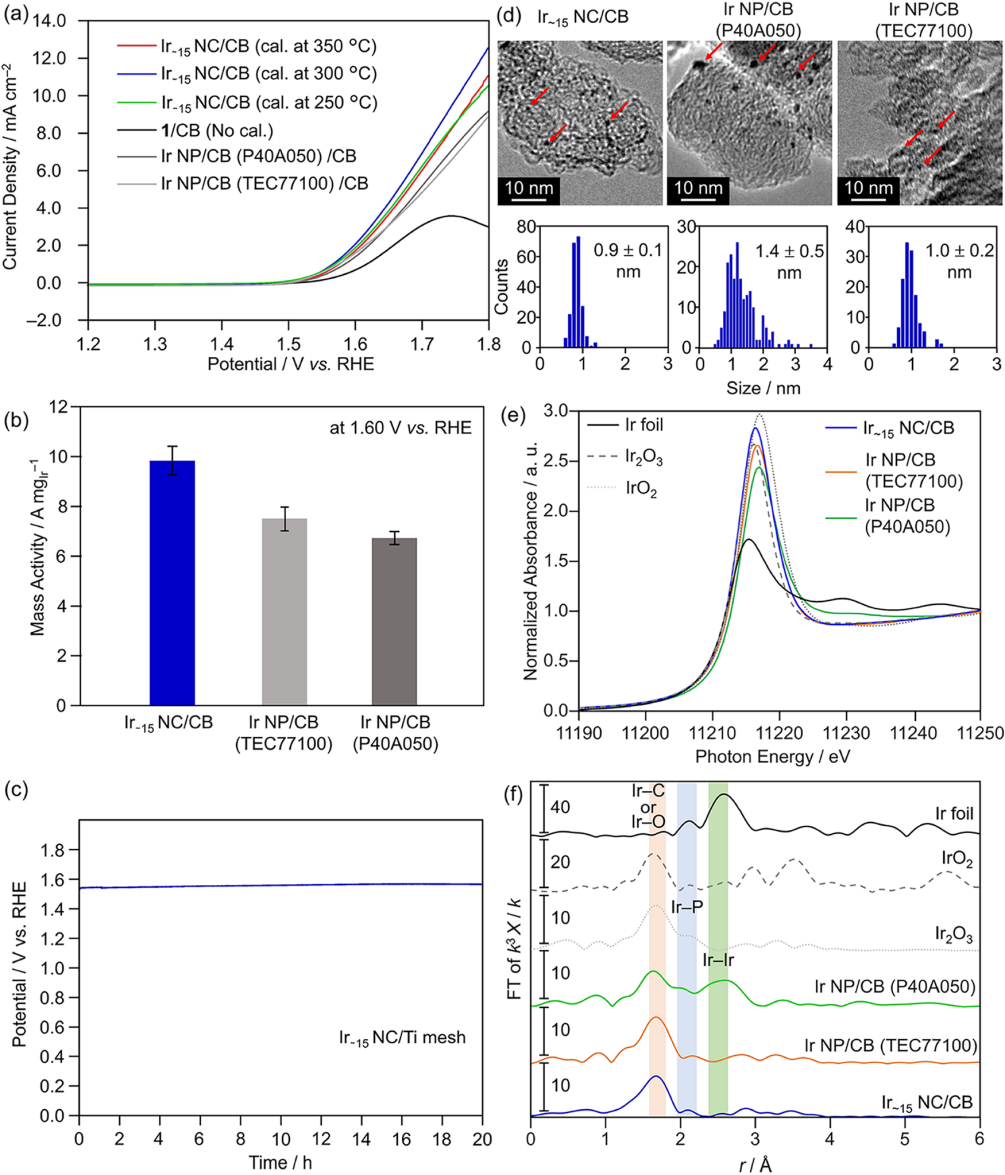
Comparison of Ir_∼15_ NC and commercial Ir NP catalyst as the OER catalysts. (a) Representative LSV curves. (b) OER mass activity of Ir-loaded catalysts. (c) Durability of Ir_∼15_ NC/Ti mesh. (d) TEM images (and the resulting size histogram). Ir L_3_-edge (e) XANES and (f) FT-EXAFS spectra of Ir_∼15_ NC/CB, commercial Ir NP/CB­(P40A050) and Ir NP/CB­(TEC77100). In (e) and (f), Ir foil, Ir_2_O_3_ and IrO_2_ powder as reference are also shown for comparison. In (e) and (f), the spectra measured at room temperature and the *k* range was set to 3.0–14.0 Å^–1^ for comparison.

Linear sweep voltammetry (LSV) was conducted to determine the mass activity at each potential, and the results are presented in Figure 5a,b. For uncalcined **1**/CB, the OER-related current density increases upon applying a potential more positive than 1.6 V vs RHE during a potential sweep from negative to positive values. Conversely, **1**/CB calcined at 250 °C (Ir_∼15_ NC/CB calcined at 250 °C; Figure 5a) exhibits an increase in OER oxidation current at a more negative potential (∼1.5 V vs RHE). Furthermore, the sample calcined at the optimal temperature of 300 °C presents OER-oxidation-current generation from the most negative potential (Figure 5a). However, **1**/CB calcined at 350 °C (Ir_∼15_ NC/CB calcined at 350 °C) showed a slight decrease in OER activity. Notably, the Ir oxidation state in each catalyst varies with calcination temperature, with higher calcination temperatures leading to a more oxidized Ir electronic state (Figure S11). Given that the Ir particle size, as determined by TEM, does not significantly change with calcination temperature (Figure S10), it is inferred that the d-band-center shift due to excessive Ir particle oxidation negatively impacts OER intermediate adsorption.^40^ In this time, the absence of Ir particle aggregation was confirmed by comparing TEM images of the catalyst before and after electrochemical measurements (Figure S14).

Subsequently, the optimized sample, calcined at 300 °C (Ir_∼15_ NC/CB), was compared with the commercial Ir NP/CB catalysts. Figure 5b shows the OER mass activity per Ir weight (1.60 V vs RHE) for each Ir catalyst. Remarkably, Ir_∼15_ NC/CB exhibits approximately 1.5-times higher OER mass activity than that of the commercial Ir NP/CB­(P40A050) catalyst. Similarly, Ir_∼15_ NC/CB outperforms another commercial Ir catalyst (TEC77100) in OER activity. It is noteworthy that the Ir loading is only 1 wt % relative to CB, a very low value. This superior activity is likely attributed to the distinct electronic and geometric structure of Ir_∼15_ NC compared with larger size of Ir NPs. Despite their smaller dimensions, the Ir_∼6_ NC-dominant catalysts (Ir_∼6_ NC/CB) showed inferior OER performance compared to Ir_∼15_ NC/CB (Figure S15), suggesting that a specific geometric/electronic ensemble is required to optimize the reaction kinetics.^57^

To further investigate the origin of high activation, particle sizes of Ir catalysts were compared using TEM images. The Ir NP/CB­(P40A050) catalyst has a particle size of 1.4 ± 0.5 nm, while Ir_∼15_ NC/CB has a size of 0.9 ± 0.1 nm, 1.6-times lower (Figure 5d). This size reduction is expected to increase the surface-to-volume ratio, thereby enhancing the number of active sites and significantly improving catalytic activity. Furthermore, the electronic and geometric structures of the Ir catalysts were analyzed using Ir L_3_-edge XANES and FT-EXAFS (Figure 5e,f). The Ir L_3_-edge XANES spectrum revealed that Ir_∼15_ NC/CB possesses a more cationic electronic state than those of commercial Ir catalysts (Table S4). In the FT-EXAFS spectrum, a relatively strong Ir–Ir bond (2.5–3.0 Å) is observed for Ir NP/CB­(P40A050) catalyst, indicating that Ir NP/CB­(P40A050) catalyst consists primarily of metallic Ir NPs, despite partial surface oxidation. The presence of Ir(0) in Ir NP/CB­(P40A050) catalyst was also confirmed by Ir 4f XPS spectra (Figure S16). In OER, it is hypothesized that the relatively cationic Ir favors OER-intermediate formation more than metallic Ir.

It is widely recognized that the OER proceeds via two distinct mechanisms: the adsorbate evolution mechanism (AEM) and the lattice oxygen oxidation mechanism (LOM).^58^ Under the AEM, it is established that catalytic activity is primarily governed by the adsorption energies of intermediates, where an optimal binding strength leads to enhanced performance. Previous studies, including DFT calculations, suggest that the miniaturization of catalysts can shift these adsorption energies toward a more favorable state through quantum size effects and structural changes.^40^ On the other hand, the fundamentally disordered or amorphous-like core structure of the fine Ir NCs suggests that the LOM may be the predominant reaction pathway.^59^ In the LOM, an increase in the covalency of the Ir–O bond reduces the electron density on the oxygen atoms, facilitating the formation of highly reactive oxyl radicals (reactive oxygen species) and lowering the energy barrier for the reaction.^60^ Indeed, Ir_∼15_ NC/CB exhibits a more cationic electronic state even compared to bulk IrO_2_ (Ir^4+^), accompanied by a contraction in the Ir–O bond length due to their ultrafine size (Figure 5e and Table S5).^60–62^ This phenomenon is inferred to result from the enhanced Ir–O covalency, which effectively reduces the electron density localized on the Ir atoms.^40^ The superior OER activity of the Ir NCs may be attributed not only to the increased electrochemically active surface area and the optimization of reaction intermediates facilitated by the cationic Ir species, but also to the promotion of the LOM pathway enabled by their disordered, amorphous-like structure. The atomistic details of these mechanisms will be further elucidated in future studies through systematic DFT calculations.^58^

The durability of the high OER activity exhibited by the Ir NCs was also assessed. To mitigate the potential degradation of the CB support, durability tests were conducted using Ir_∼15_ NC supported on titanium fibers (Ir_∼15_ NC/Ti mesh). ICP-MS confirmed adsorption of **1** onto Ti mesh with an adsorption rate approaching 100%. Chronopotentiometry was performed at a current density of 10 mA cm^–2^ using Ir_∼15_ NC/Ti mesh (Figure 5c). Notably, no increase in the applied potential was observed over 20 h, demonstrating high durability. Consistent with this, as mentioned above, the average Ir particle size, determined from TEM images of Ir_∼15_ NC/CB following electrochemical measurements, remained at 1.0 ± 0.2 nm, similar to that observed before electrochemical measurements. (Figure S14). These findings suggest that the calcination process effectively immobilizes the Ir NCs on both the CB and Ti supports, preventing aggregation under applied potential.

## Conclusion

In this study, we developed a novel synthetic method for Ir NCs with an approximate diameter of 1 nm, enabling facile air-based operation and exhibiting a narrow constituent atom distribution. Subsequently, we utilized the resulting ligand-protected Ir NCs to prepare and assess OER catalysts. The key findings are summarized below:

(1) We successfully established a systematic synthesis approach for Ir NCs with an approximate 1 nm diameter and an Ir atom distribution of ±∼2, yielding both Ir_∼6_ and Ir_∼15_ NCs.
(2) We achieved high-efficiency adsorption of Ir_∼15_ NCs onto CB, maintaining the NC size during support.
(3) Ir_∼15_ NC/CB catalyst demonstrated an OER mass activity 1.5-times greater than that of a commercial Ir NP/CB catalyst.
(4) Ir_∼15_ NC-supported Ti-mesh catalyst exhibited high durability, showing no degradation after 20 h of OER activity evaluation.

While not elaborated upon in this paper, stable multinuclear Ir NCs catalysts hold significant potential as versatile homogeneous catalysts. Furthermore, the results of this study are expected to contribute to the development of high-performance OER catalysts and the establishment of design guidelines for improved water electrolysis technology. Future research aims to elucidate the detailed geometric and electronic structures of Ir NCs and their supported catalysts through theoretical calculations,^32^ high-resolution TEM,^63^ and single-crystal X-ray diffraction.^64^

## Experimental Section

### Synthesis of 1 (Ir_∼15_ NCs)

Iridium­(III) chloride hydrate (59.7 mg) and sodium hydroxide (135 mg) were dissolved in ethylene glycol (15 mL) to prepare a reaction solution. This solution was stirred at 1000 rpm and 120 °C for 7 min using a chemical station (EYELA, PPS-CTRL1) and then cooled to room temperature using an ice bath. Then, a solution of triphenylphosphine (524.5 mg) dissolved in acetone (10 mL) was quickly added to the reaction solution, and the solution was stirred at room temperature for 60 min. A mixture of ultrapure water (>18 MΩ × cm; 12 mL) and toluene (8 mL) was added to the reaction solution, and the mixture was centrifuged (2150*g* for 2 min) to isolate the upper layer. The extracted solution was evaporated. Next, a ultrapure water/methanol mixed solvent was added, and the mixture was washed and centrifuged (2150*g* for 2 min), and the supernatant was discarded for purification. The sample was washed through an eight-step gradient using 30 mL of water/methanol mixtures per step. The solvent ratios were adjusted from 10:0 to 0:10 (v/v) as follows: 10:0, 8:2, 6:4, 4:6, 2:8, and three final washes with 0:10. Then, 5 mL of toluene was added to this Ir NC solution (crude), it was centrifuged (2150*g* for 2 min), and the supernatant was retained. This extracted solution was evaporated, the residue was dissolved in 1 mL of chloroform, methanol (30 mL) was added, the mixture was centrifuged (2150*g* for 2 min), and the supernatant was discarded. This operation was repeated three times. Then, 5 mL of toluene was added, the mixture was centrifuged (2150*g* for 2 min), and the supernatant was retained. This extracted solution was evaporated and dissolved in toluene to use in further experiments.

### Preparation of 1/CB and Ir_∼15_ NC/CB

A solution of dispersed **1** was prepared, the concentration of which was adjusted by ICP-MS (Figure S17) so that 1 wt % Ir was supported, and it was added to 100 mg of CB (VULCAN XC-72). Impregnation adsorption of **1** and CB was performed in an agate mortar. This was evacuated overnight in a desiccator (**1**/CB). Then, **1**/CB was calcined in a calcination furnace under reduced pressure (>5.0 × 10^3^ Pa) at 300 °C for 2 h (7 °C min^–1^). The obtained Ir_∼15_ NC/CB was used for catalytic tests.

### Preparation of Catalytic Slurry

Ir-loaded catalyst was added to a mixed solution consisting of ultrapure water (19.1 mL), 2-propanol (6 mL), and Nafion solution (100 μL). The resulting mixed solution was ultrasonicated in an ice–water bath for 30 min to disperse the Ir-loaded catalyst in the mixed solvent, producing a catalyst slurry. The catalyst slurry (10 μL) was cast so that it was spread over the entire surface of a glassy carbon (GC) electrode, and dried at 750 rpm for 40 min.

### Electrochemical Measurements

A three-electrode system was constructed with the GC coated with the prepared catalyst slurry as the working electrode, a Pt coil counter electrode, and a Ag/AgCl reference electrode, and electrochemical measurements were performed in a 0.1 M perchloric acid (HClO_4_) aqueous solution using a potentio/galvanostat. For the measurements, N_2_ gas was bubbled for 30 min, and then CV was performed 100 times at a scan rate of 200 mV s^–1^ in the region from 0 to 1.00 V vs RHE to clean the electrode. After the CV, LSV was performed in the region from 1.00 to 1.85 V vs RHE with the scan rate at 20 mV s^–1^. We have not performed IR compensation or background subtraction.

## Supplementary Material



## Notes

Experimental section; characterization; protocol used for the preparation of samples; curve fitting results of EXAFS; results of OER activity; MALDI-MS spectra; ESI-MS spectra; XPS spectra; XANES spectra; EXAFS spectra; TEM images; STEM images; CVs; results of DFT calculations (PDF)

## Abbreviations

(NCs): nanoclusters
(OER): oxygen evolution reaction
(NPs): nanoparticles
(PR): phosphine
(SR): thiolate
(NaOH): sodium hydroxide
(PPh_3_): triphenylphosphine
(MALDI-MS): matrix-assisted laser desorption/ionization mass spectrometry
(ESI-MS): electrospray ionization mass spectrometry
(DPTP): diphenyl­(*p*-tolyl)­phosphine
(FT-IR): Fourier transformed infrared spectroscopy
(TGA): thermogravimetric analysis
(XAFS): X-ray absorption fine structure
(XANES): X-ray absorption near-edge structure
(FT-EXAFS): FT-extended X-ray absorption fine structure
(TEM): transmission electron microscopy
(HAADF-STEM): high-angle annular dark-field scanning TEM
(CB): carbon black
(ICP-MS): inductively coupled plasma mass spectrometry
(CV): cyclic voltammetry
(LSV): linear sweep voltammetry
