# Supplementary material for: Precise Synthesis of ∼1 nm Iridium Nanoclusters as a Catalyst for Efficient Oxygen Evolution
Source: J Am Chem Soc. 2026 Jun 15;148(25):26489–98. doi: 10.1021/jacs.6c06563 (PMC13339146; doi:10.1021/jacs.6c06563)
Supplement: Supplementary file 1 [file ja6c06563_si_001.pdf]

Supporting Information

Precise Synthesis of ~1-nm Iridium Nanoclusters as a Catalyst for Efficient Oxygen Evolution

Tokuhisa Kawawaki,[1,2,*,†] Kotaro Sato,[2,†] Xiaolin Liu,[3,†] Maho Kamiyama,[1] Yamato Shingyouchi,[2] Masaki Ogami,[2] D. J. Osborn[4], Gregory F. Metha[4], De-en Jiang[3,*] and Yuichi Negishi[1,2,*]

[1]Institute of Multidisciplinary Research for Advanced Materials, Tohoku University, Katahira 2−1−1, Aoba−ku, Sendai 980−8577, Japan
[2]Carbon Value Research Center, Research Institute for Science & Technology, Tokyo University of Science, Kagurazaka, Shinjuku−ku, Tokyo 162−8601, Japan
[3]Department of Chemical and Biomolecular Engineering, Vanderbilt University, Nashville, Tennessee 37235, United States
[4]Department of Chemistry, Adelaide University, Adelaide, South Australia, 5005 Australia

[†]These authors contributed equally to this work.
Corresponding Author E-mail: tokuhisa.kawawaki.d8@tohoku.ac.jp (T. Kawawaki); de-en.jiang@vanderbilt.edu (D. Jiang); yuichi.negishi.a8@tohoku.ac.jp (Y. Negishi)

Table of Contents
Page 2–3 Additional experimental section
Page 4–6 Tables S1 to S5 (XAFS results and OER activity)
Page 7–23 Figure. S1 to S17 (MALDI-MS, ESI-MS, XPS, XANES, EXAFS spectra, TEM, STEM images, OER activity, CVs and DFT calculations)
Page 24 References (1 to 18)

S1. Experimental Section

S1.1. Chemicals

All chemicals were commercially obtained and used without further purification. Sodium hydroxide (NaOH), triphenylphosphine (PPh_3), Ir standard solution (1000 mg/L), bismuth (Bi) standard solution (1000 mg/L), ultrapure water (18.2 MΩ cm), 2-propanol, and Nafion™ were obtained from FUJIFILM Wako Pure Chemical Corporation (Osaka, Japan). 2-Phenylethanethiol (PET) was obtained from Sigma-Aldrich. Acetone, acetonitrile, dichloromethane, chloroform, ethylene glycol, perchloric acid, methanol, nitric acid, and toluene were sourced from Kanto Chemical Co., Inc. (Tokyo, Japan). Carbon black (VULCAN®XC-72) was obtained from Fuel Cell Earth. (Tokyo, Japan). Commercial Ir NPs/CB(P40A050) was obtained from Premetek. Ti mesh was obtained from Nikkotechno. Commercial Ir NPs/CB(TEC77100) was obtained from Tanaka Kikinzoku. Iridium(III) chloride hydrate ($IrCl_3 \cdot xH_2O$), diphenyl(*p*-tolyl)phosphine (DPTP) and trans-2-[3-(4-*tert*-butylphenyl)-2-methyl-2-propenylidene]malononitrile (DCTB) was obtained from Tokyo Chemical Industry Co. Ltd. (TCI, Tokyo, Japan). Alumina paste (ALUMINA POLISHINNG SUSPENSION) was from Maruto Instrument Co., Ltd. (Tokyo, Japan).

S1.2. Synthesis of 2

The synthesis method of **2** is almost the same as that of **1**. The same molar amount of DPTP (608.7 mg) was used instead of triphenylphosphine as the ligand.

S1.3. Synthesis of 3

$IrCl_3 \cdot xH_2O$ (59.7 mg) and NaOH (135 mg) were dissolved in ethylene glycol (15 ml) to prepare a reaction solution. This solution was stirred with 1000 rpm at 120 °C for 3 min using a chemical station (EYELA, PPS-CTRL1), and then cooled to room temperature using ice bath. Then, a solution of PPh_3 (524.5 mg) dissolved in acetone (10 ml) was quickly added to the reaction solution, and the solution was stirred at room temperature for 60 min. A mixture of ultrapure water (>18 MΩ × cm; 12 ml) and toluene (8 ml) was added to the reaction solution, and the mixture was centrifuged (2150 g × 2 min) to extract the upper layer. The extracted solution was evaporated. Next, a ultrapure water/methanol mixed solvent was added to this, washed, centrifuged (2150 g × 2 min), and the supernatant was discarded for purification. The sample was washed through an eight-step gradient using 30 mL of water/methanol mixtures per step. The solvent ratios were adjusted from 10:0 to 0:10 (v/v) as follows: 10:0, 8:2, 6:4, 4:6, 2:8, and three final washes with 0:10. Then, 5 ml of toluene was added to this Ir NCs solution (crude), centrifuged (2150 g × 2 min), and the supernatant was extracted. This extracted solution was evaporated, dissolved in 1 ml of chloroform, added with methanol (30 ml), centrifuged (2150 g × 2 min), and the supernatant was extracted. The extracted solution was evaporated and dissolved in toluene to use further experiments.

S1.4. Synthesis of 4

$IrCl_3 \cdot xH_2O$ (59.7 mg) and NaOH (135 mg) were dissolved in ethylene glycol (15 ml) to prepare a reaction solution. This solution was stirred (1000 rpm) at 120 °C for 30 min using a chemical station (EYELA, PPS-CTRL1), and then cooled to room temperature using ice bath. PET (268 μl) in 5 ml of toluene was then quickly added to the reaction solution, and the mixture was stirred at room temperature for 60 min. The reaction solution was mixed with ultrapure water ((>18 MΩ × cm; 15 ml) and toluene (2 ml), and the mixture was centrifuged (2150 g × 2 min) to extract the upper layer. This extracted solution was evaporated until it became viscous. A ultrapure water/methanol mixed solvent was added to this to wash, and centrifuged (2150 g × 2 min), and the supernatant was discarded and purified. The sample was washed through an eight-step gradient using 30 mL of water/methanol mixtures per step. The solvent ratios were adjusted from 10:0 to 0:10 (v/v) as follows: 10:0, 8:2, 6:4, 4:6, 2:8, and three final washes with 0:10. Approximately 5 ml of toluene was added to this crude, and centrifuged (2150 g × 2 min) to extract the supernatant.

S1.5. Preparation of $Ir_{\sim15}$ NC/Ti mesh

1 were dissolved in dichloromethane to prepare an adsorption solution, and the concentration was measured by inductively coupled plasma mass spectrometry (ICP-MS). Next, **1** were added to Ti mesh so that the amount was 0.1 wt%. The Ti mesh were left in the air for 3 days to be supported. Next, the Ti mesh loaded with $Ir_{\sim15}$ NC were used as the working electrode.

S1.6. Characterization

Matrix assisted laser desorption/ionization (MALDI)-MS spectra were recorded with a JMS-S3000 spiral time-of-flight mass spectrometer (JEOL, Tokyo, Japan) equipped with a semiconductor laser (λ = 349 nm). DCTB was used as

the MALDI matrix. To minimize NC dissociation induced by laser irradiation, the NC-to-matrix ratio was fixed at 1:1000.

Electrospray ionization MS (ESI-MS) was performed with a microTOF II reflectron time-of-flight mass spectrometer (Bruker, Massachusetts, USA).

ICP-MS was performed with an Agilent 7850c spectrometer (Agilent Technologies, Tokyo, Japan). The ICP-MS measurements were performed for the solution before mixing **1** with CB or Ti mesh to estimate the adsorbed or loaded Ir content. Specifically, the NC loading was adjusted to 1 wt% with respect to the support weight based on ICP-MS quantification. The NCs were then loaded onto the supports via either the impregnation method (for CB) or the liquid-phase adsorption method (for the Ti mesh). For the ICP-MS analysis, a Bi standard solution (1000 mg/L) was employed as an internal standard, and concentrations were determined using a pre-established external calibration curve.

Transmission electron microscopy (TEM) images were recorded with a H-9500 electron microscope (HITACHI, Tokyo, Japan) or JEM-2100 electron microscope (JEOL, Tokyo, Japan) operating at 200 kV, typically using magnification of 600 000. To address the lack of reproducibility caused by low contrast and resolution, the particle size was determined by manually measuring 200 particles without using software.

Fourier transform infrared spectroscopy (FT-IR) spectra of the product were obtained using the attenuated total reflectance (ATR) method in the region between 400 and 4000 cm^{-1} by a FT/IR-4600-ATR-PRO ONE spectrometer (JASCO, Tokyo, Japan) equipped with a DLATGS detector as the average of 50 scans at 4 cm^{-1} resolution.

The ultraviolet-visible (UV-vis) absorption spectra of products were acquired in water solution at room temperature with a V-670 spectrometer (JASCO, Tokyo, Japan).

Thermogravimetric analysis (TGA) was conducted on a TGA2000SA (Bruker, Boston, MA, USA) and at a heating rate of 5 °C/min (from room temperature to 900 °C) under N_2 atmosphere.

TEM images were recorded with a JEM-2100 electron microscope (JEOL, Tokyo, Japan) operating at 200 kV, typically using magnification of 600 000. The high-angle annular dark field scanning TEM (HAADF-STEM) images were obtained by ultra-high-resolution transmission electron microscope (The FEI Titan Themis 80−200) operating at 200 kV, with a beam convergence semi angle of 25 mrad and HAADF collection angle from 56−200 mrad. Simulated image of STEM was obtained based on the DFT-optimized structure of $Ir_{15}(CO)_{19}(PH_3)_8$ using Dr. Probe software.

Ir L_3-edge X-ray absorption fine structure (XAFS) measurements were performed at the beamline BL01B1 of the SPring-8 facility of the Japan Synchrotron Radiation Research Institute (proposal numbers 2023B1825, 2024A1698 and 2024B1592). The incident X-ray beam was monochromatized by a Si(111) double-crystal monochromator. As references, XAFS spectra of all samples were recorded in transmission mode using ionization chambers. The X-ray energies for the Ir L_3-edges were calibrated using Ir foil. The X-ray absorption near edge structure (XANES) and FT-extended X-ray absorption fine structure (FT-EXAFS) spectra were analyzed using xTunes[1] as follows. The χ spectra were extracted by subtracting the atomic absorption background using cubic spline interpolation and normalized to the edge height. The normalized data were used as the XANES spectra. The k^3-weighted χ spectra in the k range of 3.0–14.0 Å^{-1} for the Ir L_3-edge were Fourier-transformed into r space for structural analysis. The curve fitting analysis of **1** under measured at 10 K was conducted for Ir–Ir, Ir–C and Ir–P bonds over in the k range of 3.0–18.0 Å^{-1} and the r range of 1.5–3.0 Å in the Ir L_3-edge FT-EXAFS spectra. The phase shifts and backscattering amplitude functions of each bond were extracted from the simulation results for each bulk material calculated using the FEFF8 program.

S1.7. Computational details

The Ir_{15} core structure was derived from a Wulff construction[2], using Ir surface energies obtained from a database of elemental crystal surface energies[3]. The resulting Ir_{19} cluster, which represents the closest size analogue to the experimentally relevant Ir_{15} system, was truncated by removing four symmetry-equivalent corner atoms to generate the Ir_{15} core. Each Ir atom was coordinated with at least one CO ligand, and additional CO ligands were distributed uniformly over the cluster surface. PH_3 ligands were subsequently attached in an approximately uniform manner. The geometry optimization was performed using the GAUSSIAN 16 package[4]. The Perdew–Burke–Ernzerhof (PBE) functional[5] was employed for electron exchange–correlation. Grimme's D3 method[6] was used with Becke-Johnson damping for dispersion interaction[7]. The LANL2DZ effective core potential (ECP) and its associated basis set[8] were used for Ir atoms while the 6-31G(d) basis set was applied to all nonmetal atoms. Frequency calculation confirmed that the optimized structure corresponds to a true minimum.

S3. Additional Tables

Table S1. Curve fitting analysis of Ir L_3-edge EXAFS data for 1 which was measured at 10 K.

Sample	Bond	C. N. [a]	R (Å) [b]	D. W. [c]	R factor (%) [d]
Ir foil [e,f]	Ir–Ir	11.4 (2)	2.71 (1)	0.061 (85)	10.0
**1** [e,g]	Ir–Ir	2.5 (3)	2.67 (4)	0.085 (26)	6.8
	Ir–C	1.1 (3)	2.08 (6)	0.060 (54)	
	Ir–P	2.1 (4)	2.40 (7)	0.115 (55)	

The numbers in parentheses are the uncertainties, for example, 12.0 (3) represents 12.0 ± 0.3. [a] Coordination number. [b] Bond length. [c] Debye–Waller factor.

[d] R factor = $\left(\sum\left(k^3X^{\exp}(k) - k^3X^{\mathrm{fit}}(k)\right)^2\right)^{1/2} \Big/ \left(\sum\left(k^3X^{\exp}(k)\right)^2\right)^{1/2}$.

[e] Fitting k range: 3.0–18.0 Å^{-1}, [f] Fitting R Range: 2.4 Å–3.4 Å for Ir foil, [g] Fitting R Range: 1.5 Å–3.0 Å for **1**.

Table S2. Comparison of OER mass activities of each OER catalyst.

Electrocatalyst	Mass Activity	Potential	Particle Size	Catalyst Loading	Electrolyte	Ref.
Ir_{15} NC/CB	11.13 mA mg_{Ir}^{-1}	1.60 V *vs.* RHE	0.9 ± 0.1 nm	1 wt%	0.1 M $HClO_4$	this work
Ir NP/CB (P40A050)	6.469 mA mg_{Ir}^{-1}	1.60 V *vs.* RHE	1.4 ± 0.5 nm	1 wt%	0.1 M $HClO_4$	this work
Ir NP/CB (TEC77100)	6.600 mA mg_{Ir}^{-1}	1.60 V *vs.* RHE	1.0 ± 0.2 nm	1 wt%	0.1 M $HClO_4$	this work
Ir/C	22.35 mA mg Ir^{-1}	1.53 V *vs.* RHE	N/A	20 wt%	1 M PBS [a]	9
Ir(VI)-ado [b]	1.7×10^5 mA mg_{Ir}^{-1}	1.75 V	N/A	0.02 mg_{Ir} cm^{-2}	PEM water electrolyzer	10
LDH-NS@DG	10 mA cm^{-2}	1.41 V *vs.* RHE	thickness ≈ 0.6 nm	0.283 mg cm^{-2}	1 M KOH	11

[a] PBS: phosphate buffer saline, [b] Ir(VI)-ado: atomically dispersed hexavalent iridium oxide.

Since the significant difference in Ir loading density and scan rate substantially affects the impact of diffusion on mass activity, mass activity should be compared at equivalent Ir loading levels for a fair assessment.

Table S3. Comparison of OER overpotentials of each OER catalyst.

Electrocatalyst	Current	Over Potential	Particle Size	Catalyst Loading	Electrolyte	Ref.
Rh_2P/C [a]	5 mA cm^{-2}	510 mV	4.7 nm	3.7 μg_{Rh} cm^{-2}	0.5 M H_2SO_4	12
Rh/C	5 mA cm^{-2}	560 mV	3.88 nm	3.7 μg_{Rh} cm^{-2}	0.5 M H_2SO_4	12
Pt/C	5 mA cm^{-2}	630 mV	7.0 ± 0.6 nm	3.7 μg_{Pt} cm^{-2}	0.5 M H_2SO_4	12
PdP_2 NP/CB	10 mA cm^{-2}	270 mV	10 nm	6.3 wt%	1 M KOH	13
Pd NP/CB	10 mA cm^{-2}	373 mV	N/A	11.2 wt%	1 M KOH	13
PdP_2 NP/CB	10 mA cm^{-2}	277 mV	10 nm	6.3 wt	1 M PBS [b]	13
Ru–Ir nanosized-coral (Ir 6 atm%)/home-made overall water-splitting cell	10 mA cm^{-2}	170 mV	57 ± 7 nm 3 nm-thick sheets	0.15 mg cm^{-2}	0.05 M H_2SO_4	14
Ru–Ir nanosized-coral (Ir 6 atm%)	10 mA cm^{-2}	165 mV	57 ± 7 nm 3 nm-thick sheets	0.05 mg metal cm^{-2}	0.05 M H_2SO_4	14
Ir NP	10 mA cm^{-2}	371 mV	1.9 ± 0.3 nm	0.05 mg metal cm^{-2}	0.05 M H_2SO_4	14
Ru NP	10 mA cm^{-2}	550 mV	4.9 ± 0.8 nm	0.05 mg metal cm^{-2}	0.05 M H_2SO_4	14
Ru–Ir NP	10 mA cm^{-2}	242 mV	3.9 ± 0.7 nm	0.05 mg metal cm^{-2}	0.05 M H_2SO_4	14
Ir–$NiCo_2O_4$ NSs	10 mA cm^{-2}	240mV	Single atom	0.41%	0.5 M H_2SO_4	15
Ir–Co_3O_4	10 mA cm^{-2}	234mV	Single atom	1.05 atm%	0.5 M H_2SO_4	16
Ir_{sac}-NiFe LDH	50 mA cm^{-2}	213mV	Single atom	1.05 atm%	1.0 M KOH	17

[a] Rh_2P/C: rhodium phosphide electrocatalyst loaded in the form of nanocubes dispersed in high-surface-area carbon,

[b] PBS: phosphate buffer saline.

Since the significant difference in Ir loading density and scan rate substantially affects the impact of diffusion on mass activity, mass activity should be compared at equivalent Ir loading levels for a fair assessment.

Table S4. The energy position of the half-maximum of the white line ($E^{1/2}$) in the Ir L_3-edge XANES spectra for each sample.

Sample	$E^{1/2}$
Ir foil	11210.657
Ir_2O_3	11212.329
IrO_2	11212.853
**1**	11212.871
**1**/CB	11213.166
$Ir_{\sim15}$ NC/CB	11212.969
Ir NP/CB(TEC77100)	11212.871
Ir NP/CB(P40A050)	11212.510

Table S5. Curve fitting analysis of Ir L_3-edge EXAFS data for $Ir_{\sim15}$ NC/CB.

Sample	Bond	C. N. [a]	R (Å) [b]	D. W. [c]	R factor (%) [d]
Ir_2O_3	Ir−O	6.4 (3)	2.06 (3)	0.087 (30)	9.2
IrO_2	Ir−O	5.6 (2)	2.01 (3)	0.055 (27)	13.7
$Ir_{\sim15}$ NC/CB	Ir−O/C	5.2 (3)	1.99 (3)	0.072 (30)	16.5

The numbers in parentheses are the uncertainties, for example, 12.0 (3) represents 12.0 ± 0.3. [a] Coordination number. [b] Bond length. [c] Debye–Waller factor.

[d] R factor = $\left(\sum\left(k^3X^{\exp}(k) - k^3X^{\mathrm{fit}}(k)\right)^2\right)^{1/2} \Big/ \left(\sum\left(k^3X^{\exp}(k)\right)^2\right)^{1/2}$.

Fitting k range: 3.0–14.0 $Å^{-1}$ and Fitting R Range: 1.5 Å–2.0 Å.

S4. Additional Figures

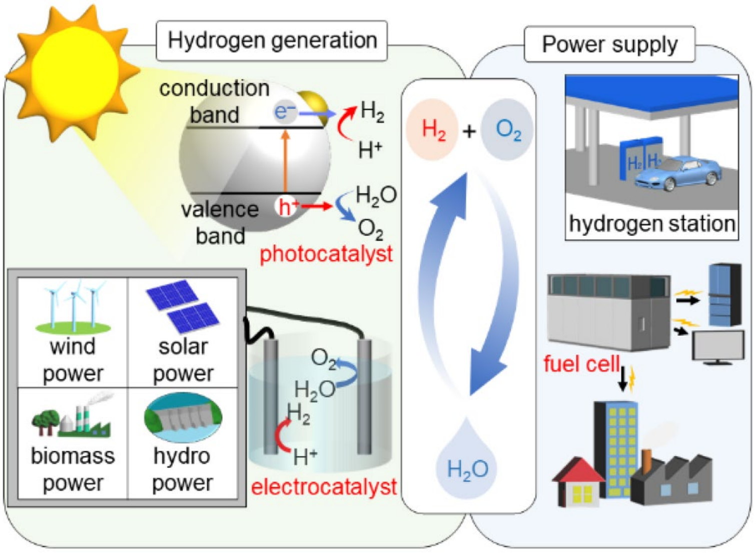

Figure S1. Conceptual diagram of an energy conversion system for a sustainable society. Schematic of the energy conversion system expected for constructing a sustainable society. Note that sunlight also produces wind, biomass, and hydro power in addition to solar power. Reproduced with permission from ref. 18. Copyright 2021 The Royal Society of Chemistry.

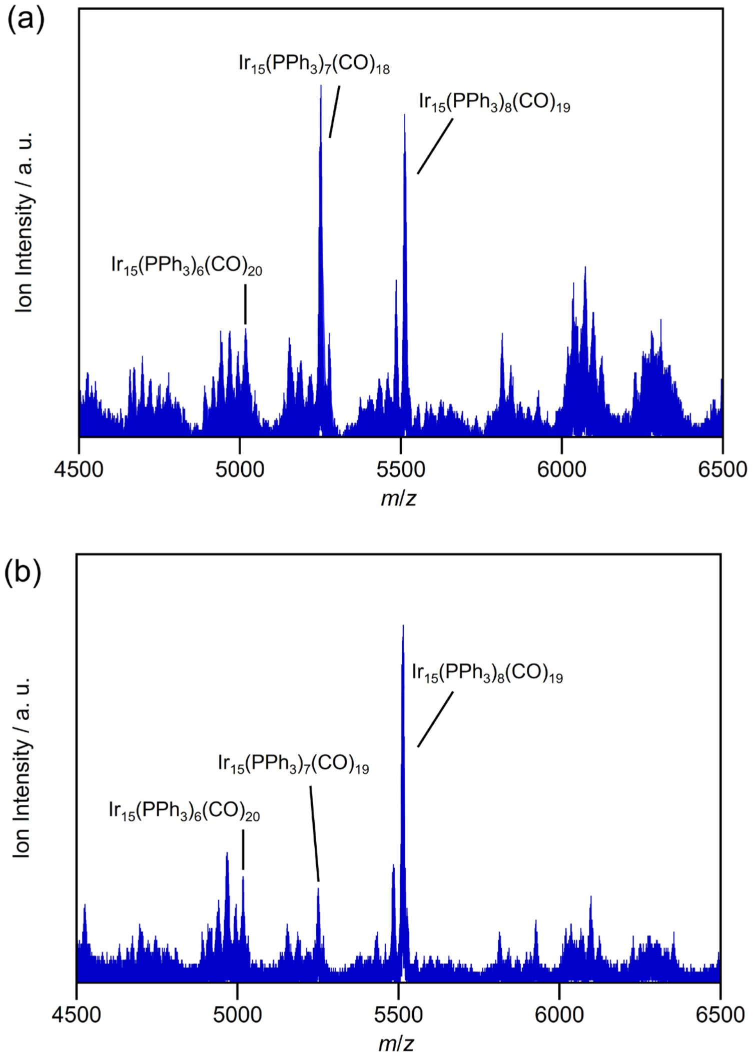

Figure S2. Characterization of 1 by mass spectrometry. ESI-MS (a) before and (b) after exposing **1** to air for one-week induced oxygen etching.

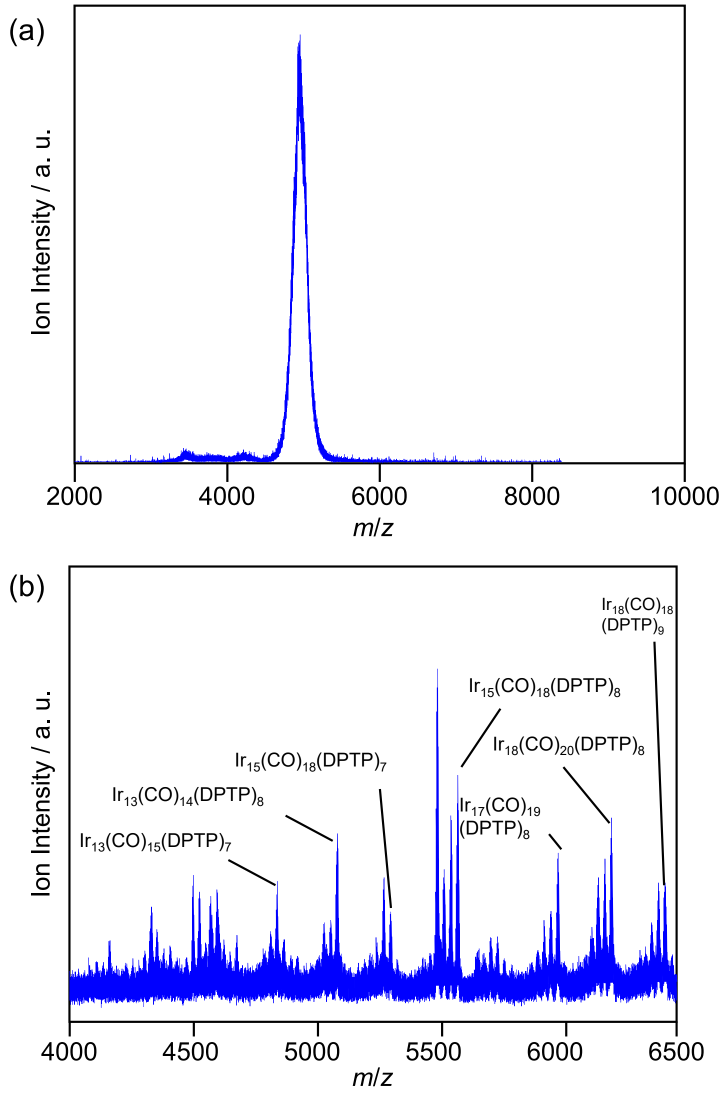

Figure S3. Characterization of 2 by mass spectrometry. (a) MALDI-MS and (b) ESI-MS spectra of **2**.

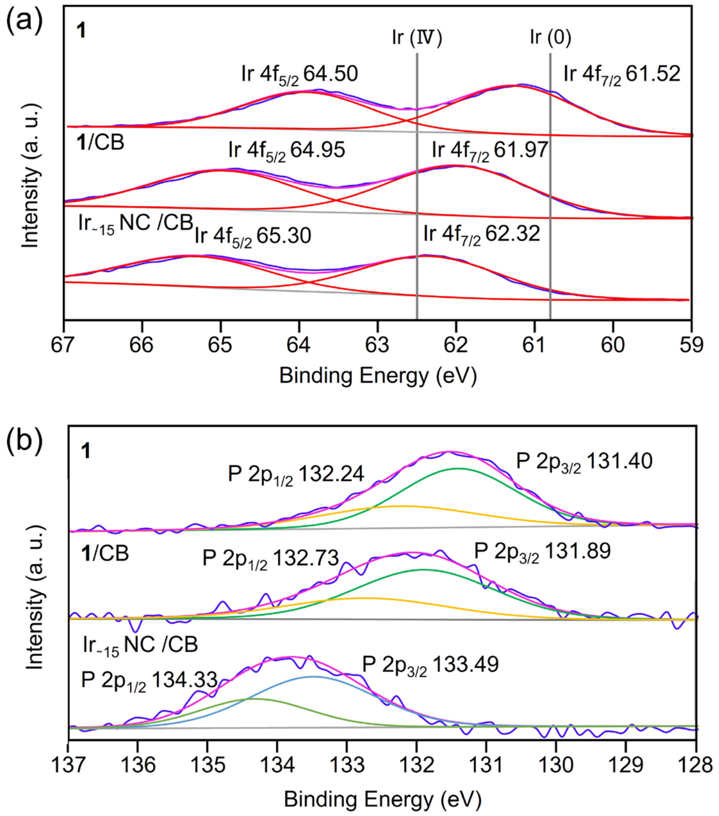

Figure S4. XPS spectra of samples. (a) Ir $4f_{7/2}$ and (b) P $2p_{3/2}$ XPS spectra (blue line) and their fitting results (red, green, and magenta lines) for **1**, **1**/CB and $Ir_{\sim 15}$ NC/CB. In (a), grey vertical lines indicate the position of Ir(0) and Ir(IV). The PPh_3 ligands coordinated to the Ir core in **1** shifted toward a more oxidized state upon support on CB, likely due to subtle structural rearrangements of the NC framework. Subsequent calcination prompted the detachment of these ligands from the Ir atoms; they remained on the CB surface in a further oxidized form, presumably as phosphine oxides ($O{=}PPh_3$, etc.).

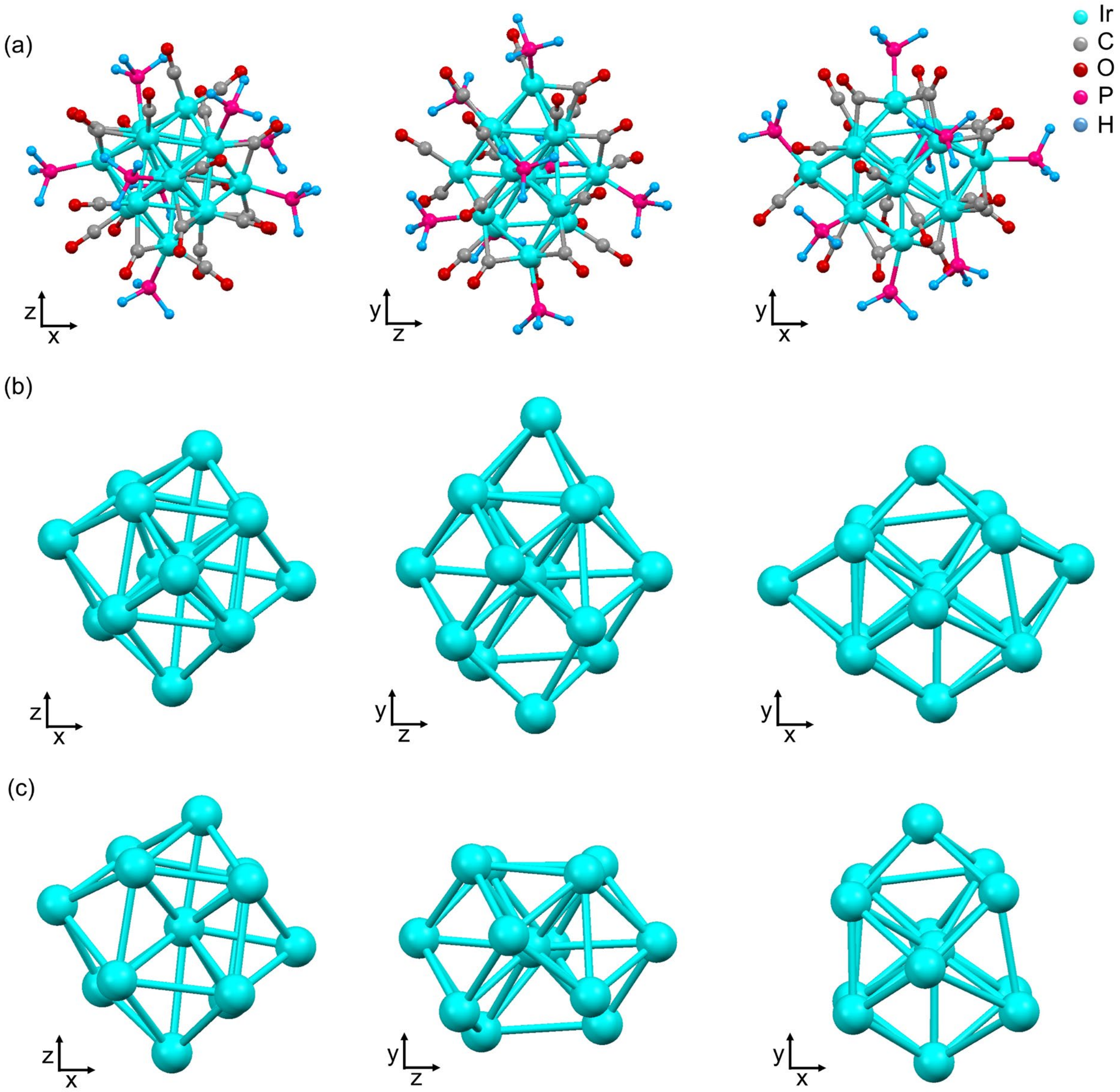

Figure S5. Geometric structure of $[Ir_{15}(CO)_{19}(PH_3)_8]^+$ determined by DFT calculation. Structure of $[Ir_{15}(CO)_{19}(PH_3)_8]^+$ viewed from different angles: (a) Total, (b) Ir_{15} kernel and (c) Ir_{13} core structure. In (b) and (c), for better visual clarity, the hydrogen, carbon, phosphine atoms have been simplified.

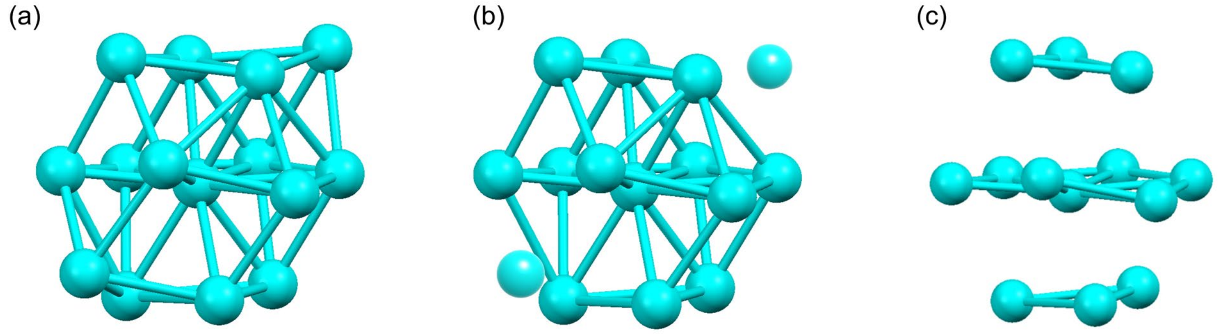

Figure S6. Geometry of core structure for $[Ir_{15}(CO)_{19}(PH_3)_8]^+$ determined by DFT calculation. Structure of (a) total and (b) structurally isolated Ir_{15} kernel and (c) Ir_{13} core in $[Ir_{15}(CO)_{19}(PH_3)_8]^+$. For better visual clarity, the hydrogen, carbon, phosphine atoms have been simplified.

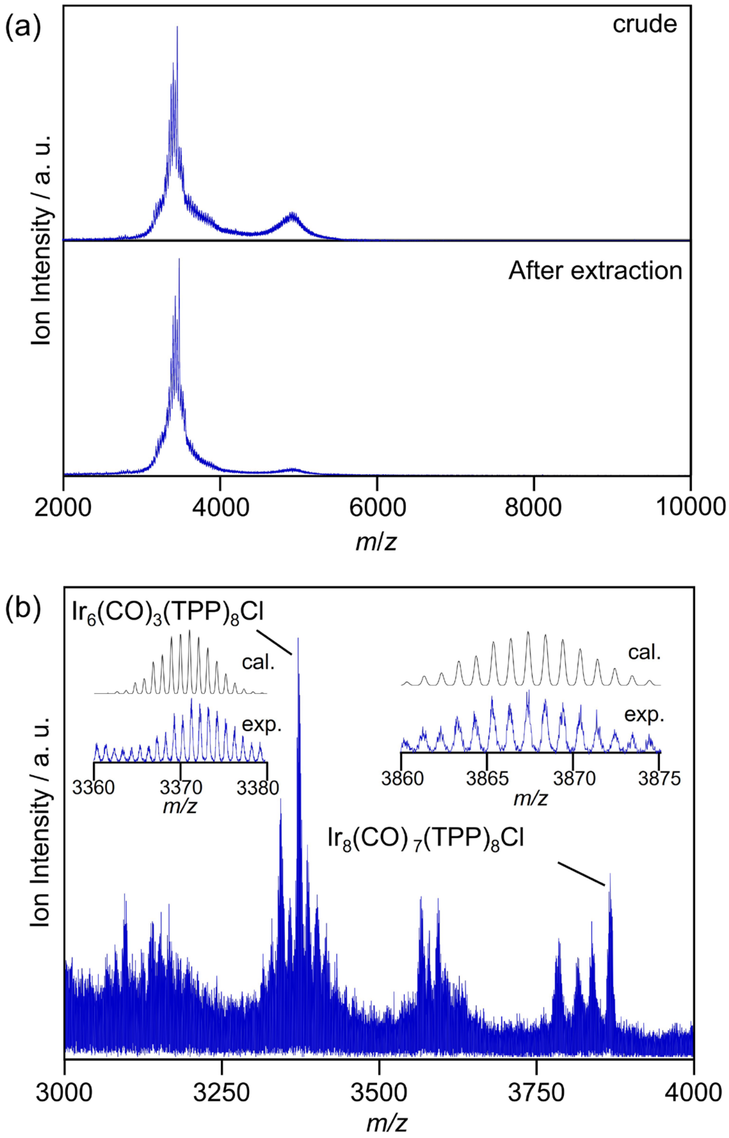

Figure S7. Characterization of 3 by mass spectrometry. (a) MALDI-MS and (b) ESI-MS spectra of **3**.

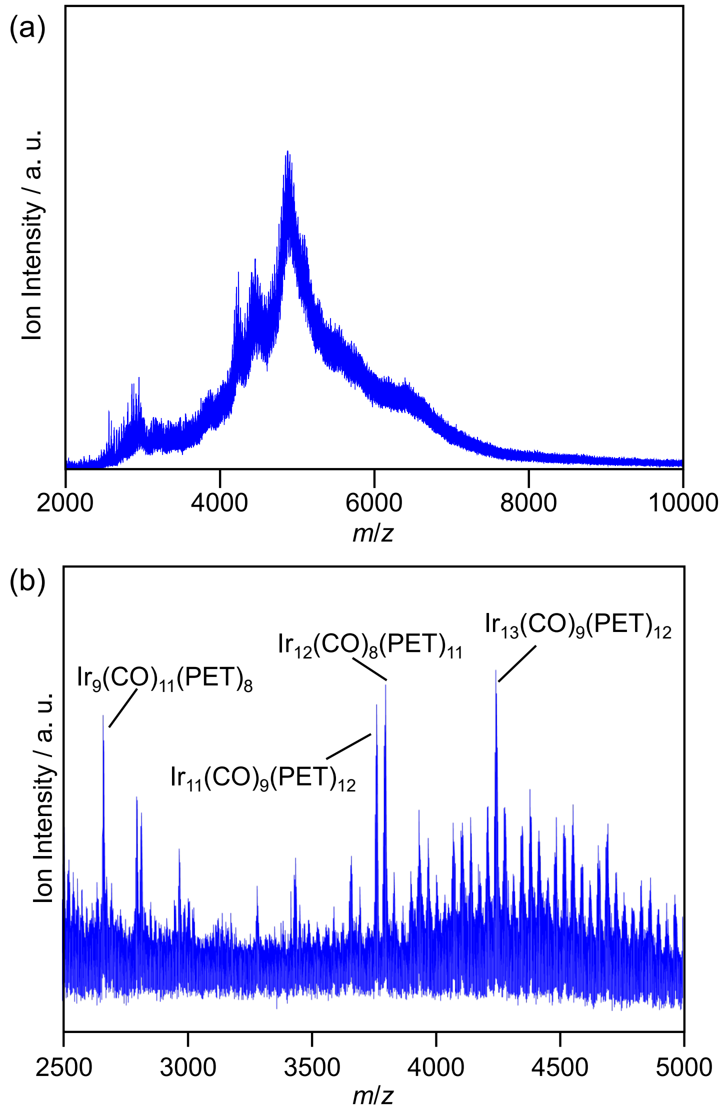

Figure S8. Characterization of 4 by mass spectrometry. (a) MALDI-MS and (b) ESI-MS spectra of **4**.

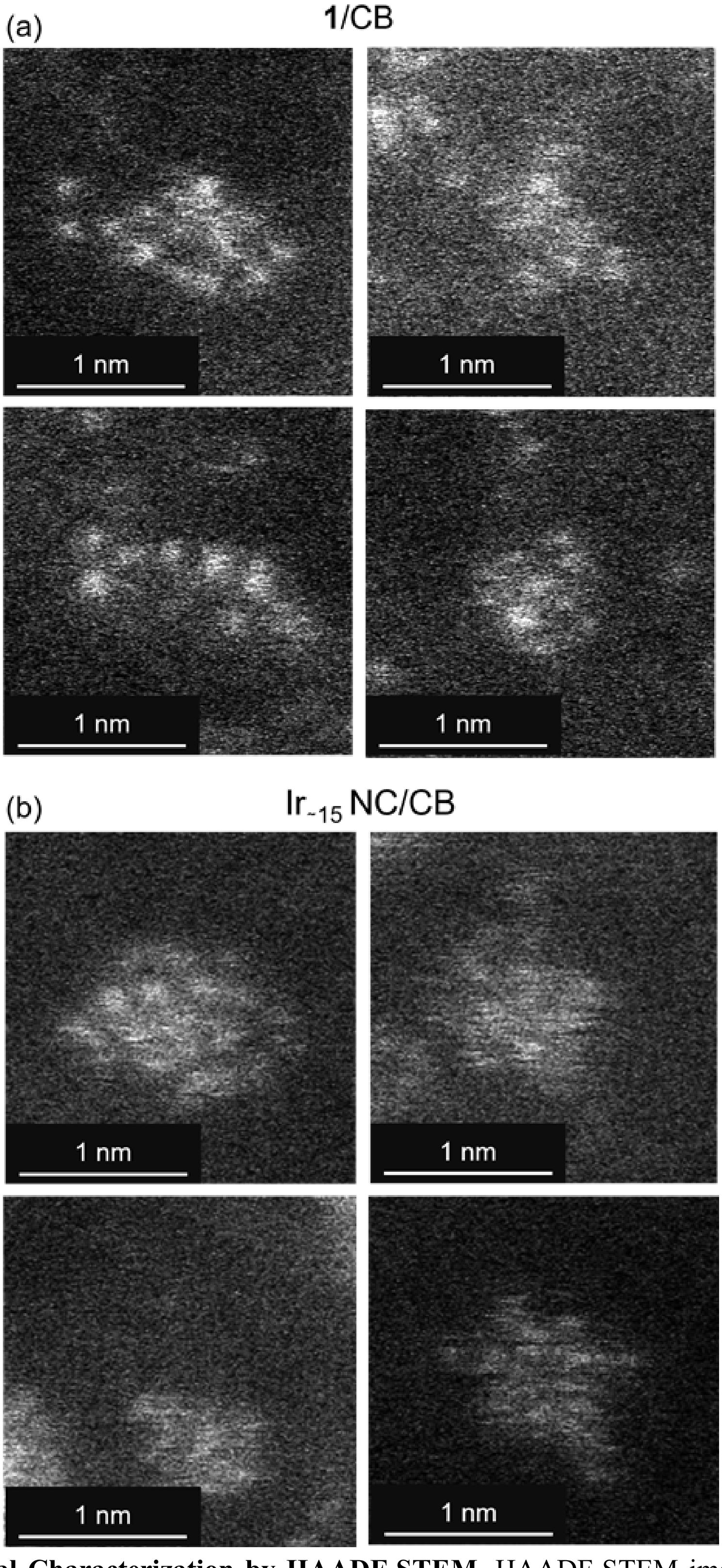

Figure S9. Structural Characterization by HAADF-STEM. HAADF-STEM images of (a) $Ir_{\sim15}$ NC-supported catalysts before (**1**/CB) and (b) after calcinated at 300 °C ($Ir_{\sim15}$ NC/CB).

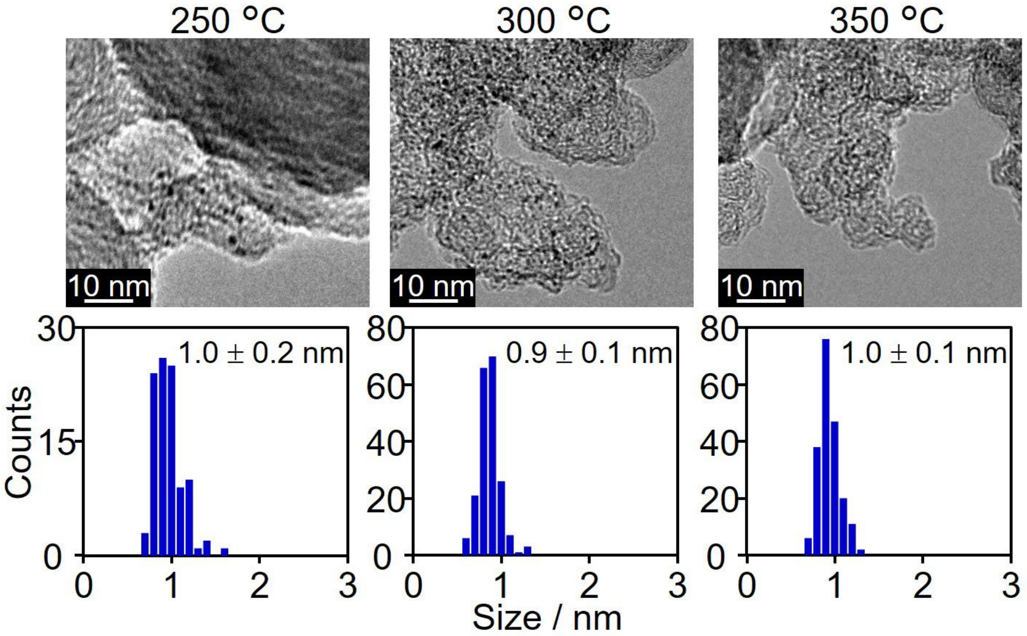

Figure S10. Effect of calcination temperature on the size of Ir$_{\sim15}$ NC/CB. TEM images and the resulting size histogram for calcinated **1**/CB at 250 °C (Ir$_{\sim15}$ NC/CB cal. at 250°C), 300 °C (Ir$_{\sim15}$ NC/CB) and 350 °C (Ir$_{\sim15}$ NC/CB cal. at 350°C).

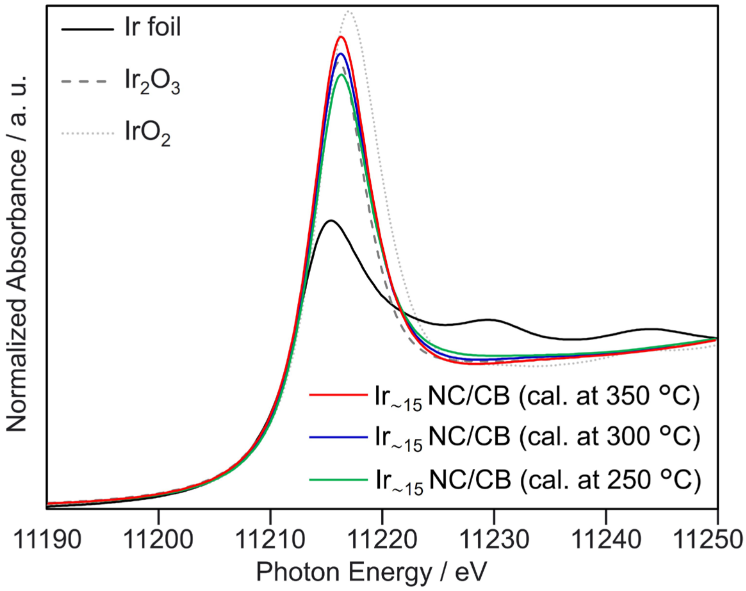

Figure S11. Electronic state of Ir at different calcination temperatures. Ir L_3-edge XANES spectra for Ir particles for calcinated **1**/CB at 250 °C ($Ir_{\sim15}$ NC/CB cal. at 250 °C), 300 °C ($Ir_{\sim15}$ NC/CB. at 300 °C) and 350 °C ($Ir_{\sim15}$ NC/CB cal. at 350 °C). Ir foil, Ir_2O_3, and IrO_2 powder as references are also shown for comparison.

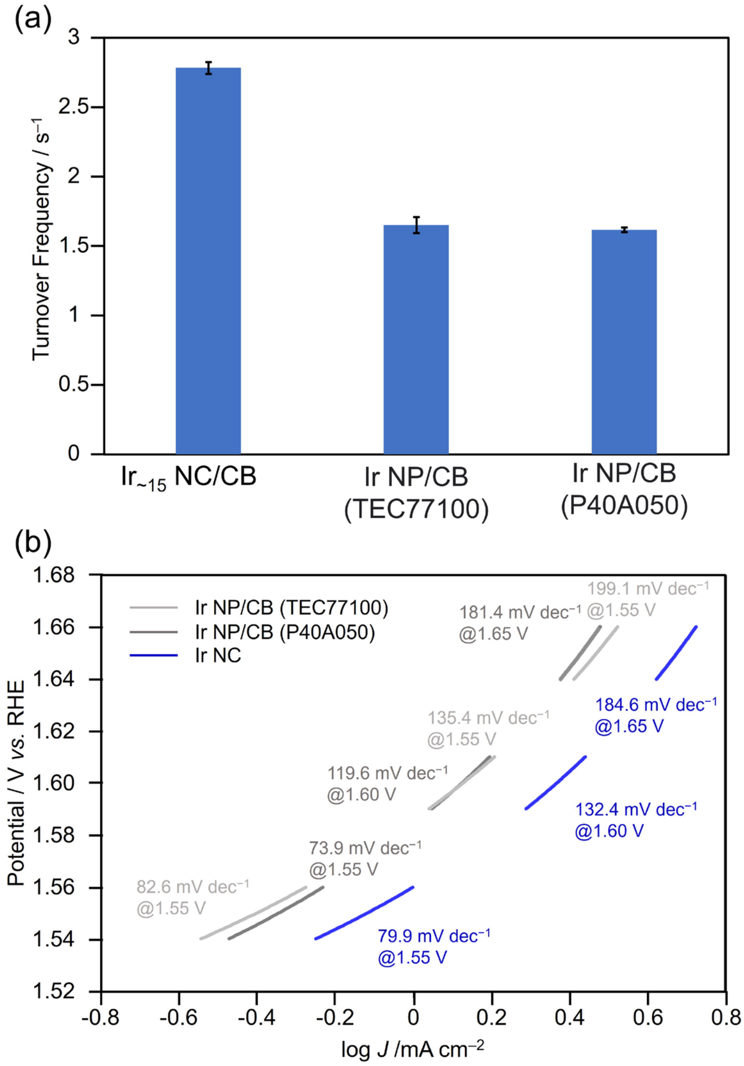

Figure S12. Turnover frequency and Tafel plot for OER activity. (a) Turnover frequency at 1.60 V vs. RHE and (b) Tafel plot at 1.55, 1.60 and 1.65 V vs. RHE for Ir-loaded catalysts.

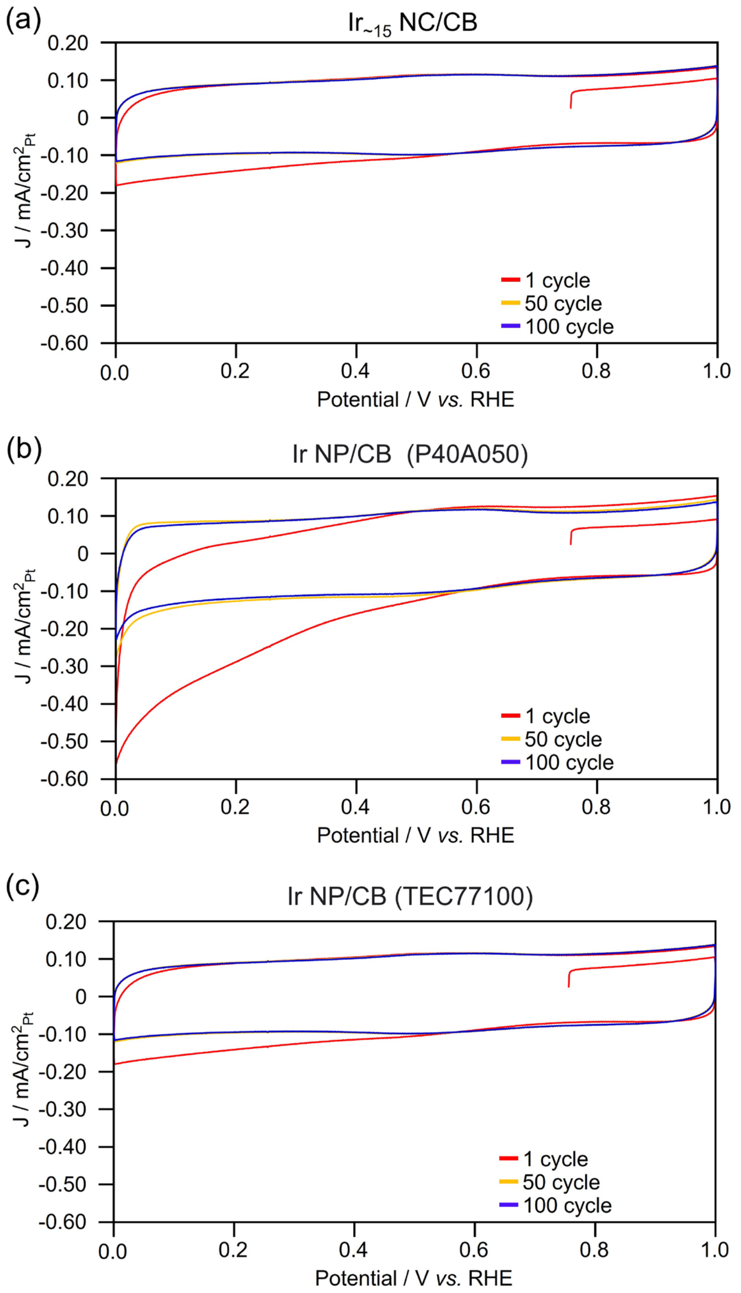

Figure S13. Results of cyclic voltammetry measurements. CV results at 1, 50, and 100 cycles obtained by performing CV measurements in the range of 0 to 1.00 V vs. RHE at a scan rate of 200 mV s^{-1} to clean the electrode for (a) $Ir_{\sim 15}$ NC/CB, (b) Ir NP/CB(P40A050) and (c) Ir NP/CB(TEC77100).

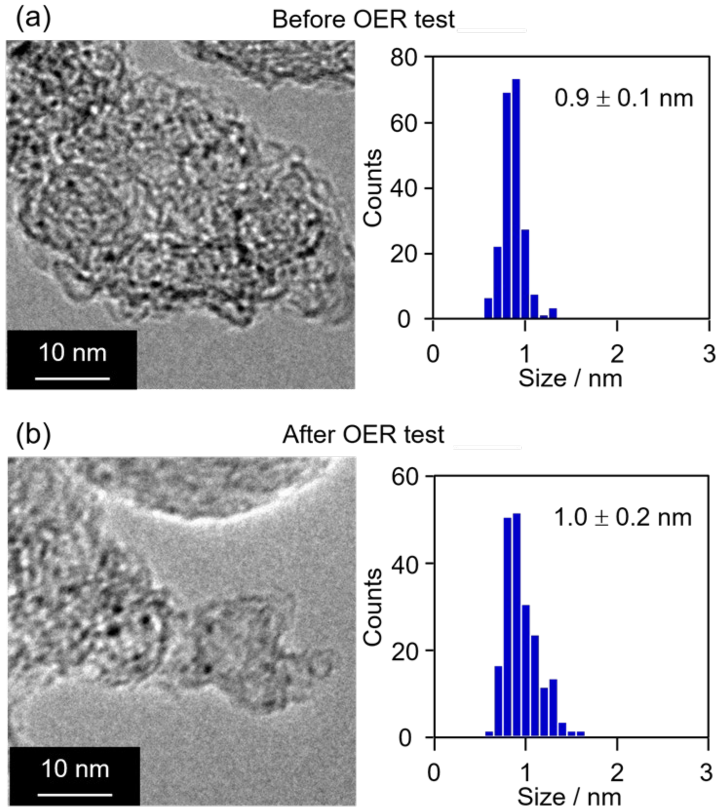

Figure S14. Change in particle size of $Ir_{\sim 15}$ NC/CB before and after OER measurement. TEM images and the resulting size histogram of $Ir_{\sim 15}$ NC/CB (a) before and (b) after OER catalytic measurements.

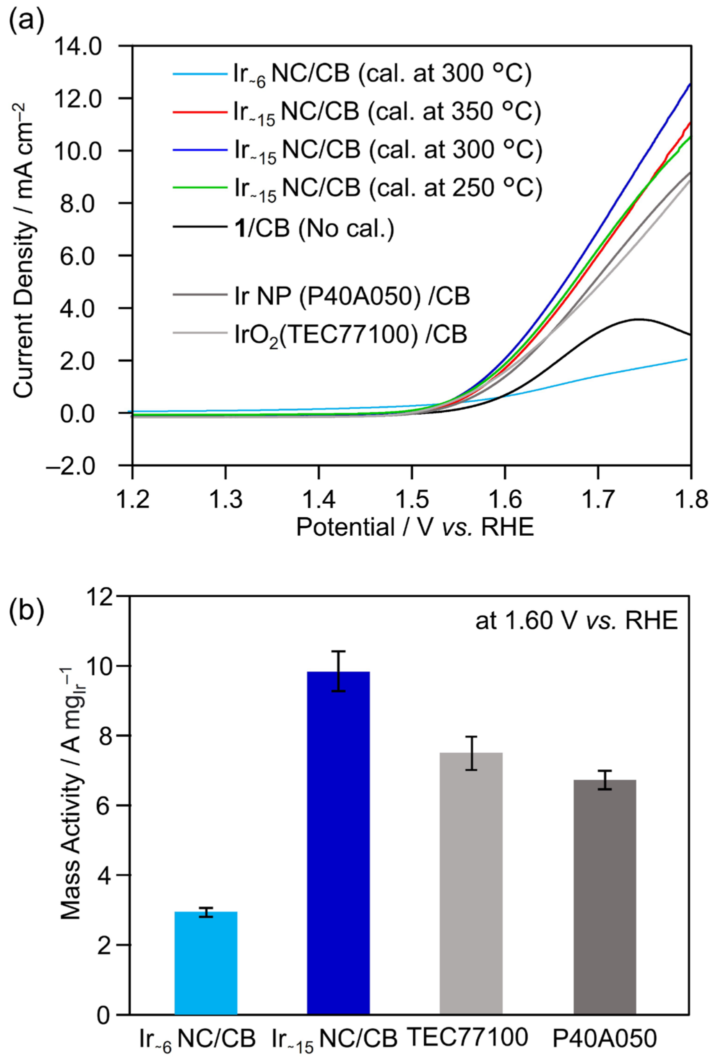

Figure S15. Comparison of Ir~6 NC and other Ir catalyst as the OER catalysts. (a) Representative LSV curves and (b) OER mass activity of Ir-loaded catalysts.

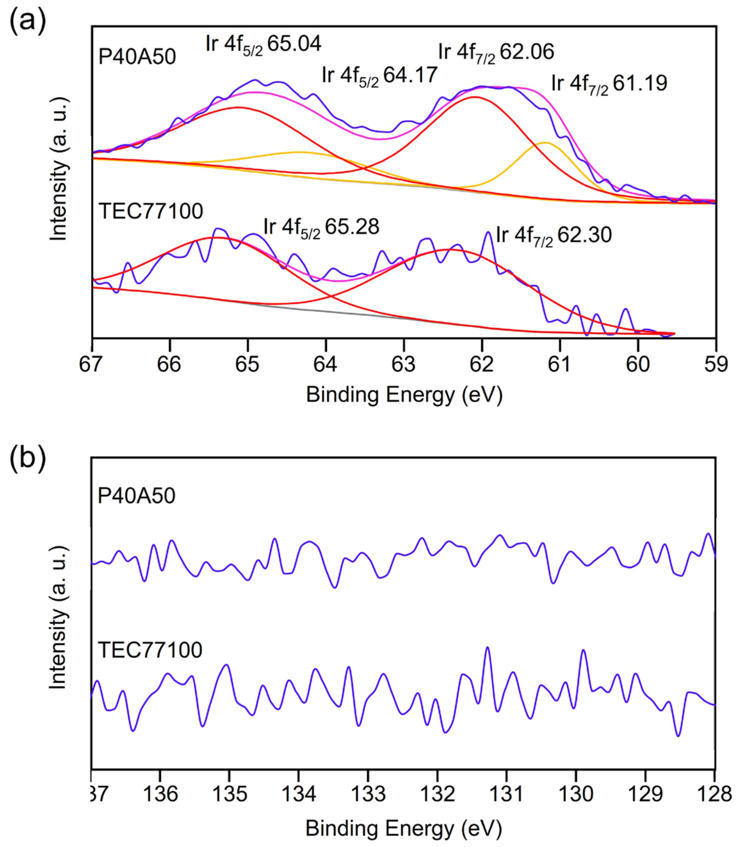

Figure S16. XPS spectra of commercial catalysts. (a) Ir $4f_{7/2}$ and (b) P $2p_{3/2}$ XPS spectra (blue line) and their fitting results (red, yellow and magenta lines) for Ir NP/CB(P40A050) and Ir NP/CB(TEC77100).

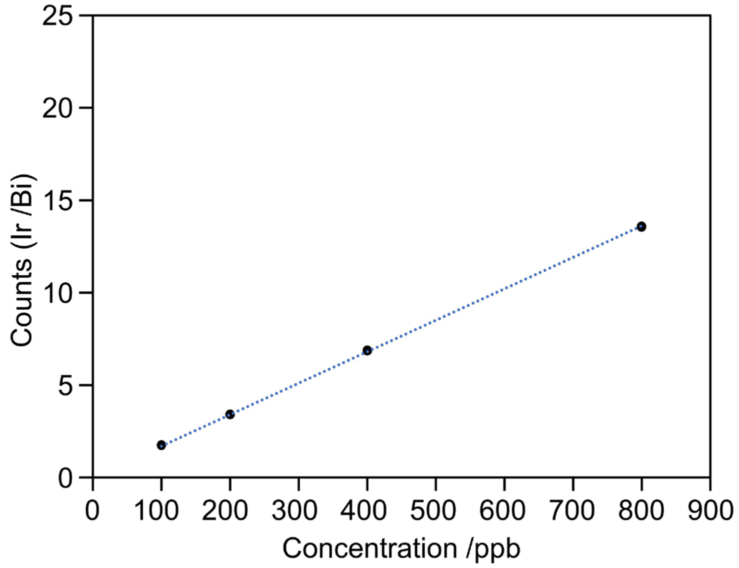

Figure S17. Calibration curve data for ICP-MS measurements.

S5. References

(1) Asakura, H.; Yamazoe, S.; Misumi, T.; Fujita, A.; Tsukuda, T.; Tanaka, T. Xtunes: A New Xas Processing Tool for Detailed and on-the-Fly Analysis. *Radiat. Phys. Chem.* **2020**, *175*, 108270.

(2) Chatzigoulas, A.; Karathanou, K.; Dellis, D.; Cournia, Z. NanoCrystal: A Web-Based Crystallographic Tool for the Construction of Nanoparticles Based on Their Crystal Habit. *J. Chem. Inf. Model.* **2018**, *58* (12), 2380–2386.

(3) Tran, R.; Xu, Z.; Radhakrishnan, B.; Winston, D.; Sun, W.; Persson, K. A.; Ong, S. P. Surface Energies of Elemental Crystals. *Sci. Data* **2016**, *3*, 160080.

(4) M. J. Frisch, G. W. Trucks, H. B. Schlegel, G. E. Scuseria, M. A. Robb, J. R. Cheeseman, G. Scalmani, V. Barone, G. A. Petersson, H. Nakatsuji, X. Li, M. Caricato, A. V. Marenich, J. Bloino, B. G. Janesko, R. Gomperts, B. Mennucci, H. P. Hratchian, J. V. Ortiz, A. F. Izmaylov, J. L.Sonnenberg, D. Williams-Young, F. Ding, F. Lipparini, F. Egidi, J. Goings, B. Peng, A. Petrone, T. Henderson, D. Ranasinghe, V. G. Zakrzewski, J. Gao, N. Rega, G. Zheng, W. Liang, M. Hada, M. Ehara, K. Toyota, R. Fukuda, J. Hasegawa, M. Ishida, T. Nakajima, Y. Honda, O. Kitao, H. Nakai, T. Vreven, K. Throssell, J. A. Montgomery, Jr., J. E. Peralta, F. Ogliaro, M. J. Bearpark, J. J. Heyd, E. N. Brothers, K. N. Kudin, V. N. Staroverov, T. A. Keith, R. Kobayashi, J. Normand, K. Raghavachari, A. P. Rendell, J. C. Burant, S. S. Iyengar, J. Tomasi, M. Cossi, J. M. Millam, M. Klene, C. Adamo, R. Cammi, J. W. Ochterski, R. L. Martin, K. Morokuma, O. Farkas, J. B. Foresman, and D. J. Fox. Gaussian 16 Revision B.01. Gaussian Inc. Wallingford CT 2016.

(5) Perdew, J. P.; Burke, K.; Ernzerhof, M. Generalized Gradient Approximation Made Simple. *Phys. Rev. Lett.* **1996**, *77* (18), 3865–3868.

(6) Grimme, S.; Antony, J.; Ehrlich, S.; Krieg, H. A Consistent and Accurate *Ab Initio* Parametrization of Density Functional Dispersion Correction (DFT-D) for the 94 Elements H-Pu. *J. Chem. Phys.* **2010**, *132* (15), 154104.

(7) Grimme, S.; Ehrlich, S.; Goerigk, L. Effect of the Damping Function in Dispersion Corrected Density Functional Theory. *J Comput Chem* **2011**, *32* (7), 1456–1465.

(8) Hay, P. J.; Wadt, W. R. *Ab Initio* Effective Core Potentials for Molecular Calculations. Potentials for the Transition Metal Atoms Sc to Hg. *J. Chem. Phys.* **1985**, *82* (1), 270–283.

(9) Wang, Q.; Xu, C.-Q.; Liu, W.; Hung, S.-F.; Bin Yang, H.; Gao, J.; Cai, W.; Chen, H. M.; Li, J.; Liu, B. Coordination Engineering of Iridium Nanocluster Bifunctional Electrocatalyst for Highly Efficient and pH-Universal Overall Water Splitting. *Nat. Commun.* **2020**, *11*, 4246.

(10) Li, A.; Kong, S.; Adachi, K.; Ooka, H.; Fushimi, K.; Jiang, Q.; Ofuchi, H.; Hamamoto, S.; Oura, M.; Higashi, K.; Kaneko, T.; Uruga, T.; Kawamura, N.; Hashizume, D.; Nakamura, R. Atomically Dispersed Hexavalent Iridium Oxide from MnO_2 Reduction for Oxygen Evolution Catalysis. *Science* **2024**, *384*, 666-670.

(11) Jia, Y.; Zhang, L.; Gao, G.; Chen, H.; Wang, B.; Zhou, J.; Soo, M. T.; Hong, M.; Yan, X.; Qian, G.; Zou, J.; Du, A.; Yao, X. A Heterostructure Coupling of Exfoliated Ni–Fe Hydroxide Nanosheet and Defective Graphene as a Bifunctional Electrocatalyst for Overall Water Splitting. *Adv. Mater.* **2017**, *29*, 1700017.

(12) Duan, H.; Li, D.; Tang, Y.; He, Y.; Ji, S.; Wang, R.; Lv, H.; Lopes, P. P.; Paulikas, A. P.; Li, H.; Mao, S. X.; Wang, C.; Markovic, N. M.; Li, J.; Stamenkovic, V. R.; Li, Y. High-Performance Rh_2P Electrocatalyst for Efficient Water Splitting. *J. Am. Chem. Soc.* **2017**, *139*, 5494-5502.

(13) Luo, F.; Zhang, Q.; Yu, X.; Xiao, S.; Ling, Y.; Hu, H.; Guo, L.; Yang, Z.; Huang, L.; Cai, W.; Cheng, H. Palladium Phosphide as a Stable and Efficient Electrocatalyst for Overall Water Splitting. *Angew. Chem. Int. Ed.* **2018**, *57*, 14862-14867.

(14) Wu, D.; Kusada, K.; Yoshioka, S.; Yamamoto, T.; Toriyama, T.; Matsumura, S.; Chen, Y.; Seo, O.; Kim, J.; Song, C.; Hiroi, S.; Sakata, O.; Ina, T.; Kawaguchi, S.; Kubota, Y.; Kobayashi, H.; Kitagawa, H. Efficient Overall Water Splitting in Acid with Anisotropic Metal Nanosheets. *Nat. Commun.* **2021**, *12*, 1145.

(15) Yin, J.; Jin, J.; Lu, M.; Huang, B.; Zhang, H.; Peng, Y.; Xi, P.; Yan, C.-H. Iridium Single Atoms Coupling with Oxygen Vacancies Boosts Oxygen Evolution Reaction in Acid Media. *J. Am. Chem. Soc.* **2020**, *142*, 18378-18386.

(16) Zhu, Y.; Wang, J.; Koketsu, T.; Kroschel, M.; Chen, J. M.; Hsu, S. Y.; Henkelman, G.; Hu, Z.; Strasser, P.; Ma, J. Iridium Single Atoms Incorporated in Co_3O_4 Efficiently Catalyze the Oxygen Evolution in Acidic Conditions. *Nat. Commun.* **2022**, *13*, 7754.

(17) Duan, Z.; Cui, Z.; Gao, Z.; Xu, W.; Liang, Y.; Jiang, H.; Li, Z.; Wang, F.; Zhu, S. Single-Atom Iridium Orchestrates a Reaction Pathway Shift to Activate Lattice Oxygen for Efficient Oxygen Evolution. *ACS Catal.* **2025**, *15*, 16882-16892.

(18) Kawawaki, T.; Kataoka, Y.; Ozaki, S.; Kawachi, M.; Hirata, M.; Negishi, Y. Creation of Active Water-Splitting Photocatalysts by Controlling Cocatalysts Using Atomically Precise Metal Nanoclusters. *Chem. Commun.* **2021**, *57*, 417-440.
